# Harnessing Solar‐Driven Photothermal Effect toward the Water–Energy Nexus

**DOI:** 10.1002/advs.201900883

**Published:** 2019-07-22

**Authors:** Chao Zhang, Hong‐Qing Liang, Zhi‐Kang Xu, Zuankai Wang

**Affiliations:** ^1^ Department of Mechanical Engineering City University of Hong Kong Hong Kong China; ^2^ Carbon Dioxide Activation Center (CADIAC) Interdisciplinary Nanoscience Center (iNANO) and Department of Chemistry Aarhus University 8000 Aarhus C Denmark; ^3^ MOE Key Laboratory of Macromolecular Synthesis and Functionalization Department of Polymer Science and Engineering Zhejiang University Hangzhou 310027 China

**Keywords:** interfacial evaporation, low‐energy desalination, photothermal effect, solar energy, water harvesting, water–energy nexus

## Abstract

Producing affordable freshwater has been considered as a great societal challenge, and most conventional desalination technologies are usually accompanied with large energy consumption and thus struggle with the trade‐off between water and energy, i.e., the water–energy nexus. In recent decades, the fast development of state‐of‐the‐art photothermal materials has injected new vitality into the field of freshwater production, which can effectively harness abundant and clean solar energy via the photothermal effect to fulfill the blue dream of low‐energy water purification/harvesting, so as to reconcile the water–energy nexus. Driven by the opportunities offered by photothermal materials, tremendous effort has been made to exploit diverse photothermal‐assisted water purification/harvesting technologies. At this stage, it is imperative and important to review the recent progress and shed light on the future trend in this multidisciplinary field. Here, a brief introduction of the fundamental mechanism and design principle of photothermal materials is presented, and the emerging photothermal applications such as photothermal‐assisted water evaporation, photothermal‐assisted membrane distillation, photothermal‐assisted crude oil cleanup, photothermal‐enhanced photocatalysis, and photothermal‐assisted water harvesting from air are summarized. Finally, the unsolved challenges and future perspectives in this field are emphasized. It is envisioned that this work will help arouse future research efforts to boost the development of solar‐driven low‐energy water purification/harvesting.

## 1 Introduction

Freshwater scarcity has become a daunting challenge as the increase of ever‐growing population and economic development.^1^, ^2^ Currently, there are still over 1.6 billion people living in water‐stressed areas without access to clean and safe drinking water, and such a situation will grow much worse in the coming decades.^3^ In spite of the earth with abundant water resource, the potable fresh water only accounts for around 2.5%,^4^ and its fraction has been remarkably declining due to the frequent droughts and severe water pollutions. In order to address the issue of global water problem, considerable efforts have been devoted to developing various advanced materials and techniques for obtaining high‐quality freshwater from brines or even polluted water. Up to date, most of the existing water purification plants have adopted reverse osmosis (RO) or low‐temperature multiple‐effect distillation (MED) technologies.^5^, ^6^, ^7^, ^8^ However, they are susceptible to formidable drawbacks such as high energy consumption (i.e., 5 and 8 kWh m^−3^ for RO and MED, respectively) and the inevitable need of large centralized infrastructure, which greatly limit their practical applications, especially in the offshore areas, small villages, or remote off‐grid regions. Therefore, it is highly desired to develop an appealing solution to overcome the trade‐off between energy consumption and water purification productivity. From the practical perspective, the ideal next‐generation water purification technology requires to effectively solve the water–energy nexus and possess these traits as follows: low‐energy, low‐cost, easy‐to‐implement, small cubage and portability, scalable manufacturing system, and high productivity.

As a kind of abundant, clean, and renewable energy, solar energy has received much research interest as the promising alternative to conventional energy such as fossil fuels. Motivated by the demands of both fundamental researches and practical applications, much effort has been paid to harness solar energy for diverse ever‐evolving applications ranging from power generation,^9^, ^10^ photocatalysis/catalysis,^11^, ^12^, ^13^, ^14^ solar cells,^15^, ^16^ and water purification^14^, ^17^, ^18^ to water desalination.^19^, ^20^, ^21^ Among them, photothermal effect has been widely utilized because of their superior ability to convert solar energy into thermal energy. This unique self‐heating feature can not only enhance the intrinsic properties of materials, but also endow materials with some extraordinary functions. Despite great promise offered by photothermal effect as a powerful tool to reduce energy consumption, photothermal materials usually struggle to low conversion efficiency and thus exhibit modest utilization efficiency of solar energy. It has been well known that the power distribution of solar radiation on the earth surface is divided into three parts as follows: ≈7% for the ultraviolet region (300–400 nm), ≈43% for the visible region (400– 700 nm), and ≈50% for the near‐infrared region (700–2500 nm).^22^ Therefore, in aim to make the most of solar energy, an inevitable challenge is how to design and exploit strong and broadband solar absorbers, covering the full solar spectrum range from 300 to 2500 nm.

Thanks to the emergence of various state‐of‐the‐art photothermal materials in the past decade, great progress has been made in the design and development of solar‐driven water purification/harvesting technologies. Given that most of the regions with high water shortage have abundant solar energy resources, these emerging photothermal applications show great potential to utilize solar energy to directly solve the issue of water shortage and improve the overall resilience of the water–energy nexus. To date, two typical approaches have been studied to produce clean water under the aid of solar energy: a) extracting freshwater from brines or polluted water (i.e., desalination or removing contaminants) and b) collecting freshwater from air (i.e., even low humidity environment).^23^, ^24^ First, solar‐driven photothermal effect can be directly used or integrated into other water purification technologies. For example, solar‐generated thermal energy has the capability of remarkably accelerating water evaporation, as well as acting as the hot source during conventional membrane distillation (MD) for seawater desalination. Besides, photocatalysis and physical adsorption process are also enhanced by solar‐driven photothermal effect to fast remove contaminants within water (e.g., dye and crude oil). Second, some strong water‐sorption materials can be employed to harvest freshwater from air or humidity environment powered by solar energy. The role of solar‐driven photothermal effect is to provide low‐grade heat for fast release of captured water within water‐sorption materials. This proof‐of‐concept technology shows great potential because the water content (i.e., vapor and droplets) of atmosphere accounts for about 10% of all other freshwater resources. Among aforementioned all photothermal applications, the interface engineering, especially the interface between solid photothermal materials and aqueous solutions, is also very crucial for deciding photothermal properties and performance of everything from evaporators to sorbents and membranes.^25^ For example, the solar evaporator tends to need a hydrophilic interface for water supply, whereas both photothermal MD and crude oil cleanup technologies require a hydrophobic interface toward targeted aqueous solutions. Thus, rational design of photothermal materials/devices shows a glimmer of hope to solve the issue of water shortage, especially in remote water‐ and energy‐stressed areas.

**Figure 1:**
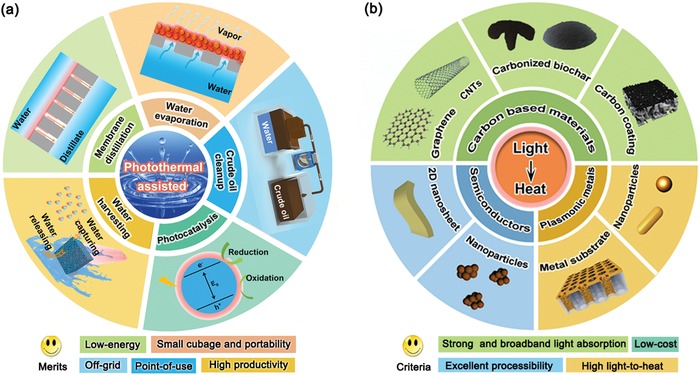
a) Emerging solar‐driven photothermal‐assisted applications for low‐energy water purification/harvesting. b) Schematic diagram of various photothermal materials.

To date, there have been many reviews related to the photothermal materials and their potential applications.^23^, ^26^, ^27^, ^28^, ^29^ However, these previously published review articles are limited to the specific topic of solar steam generation, and thus there is still no comprehensive review focusing on the latest progress and future development trend of all the solar‐driven low‐energy water purification/harvesting technologies. Given significant achievements made in these technologies beyond solar steam generation, especially photothermal‐assisted MD, crude oil cleanup, and water harvesting from air, it is thus time to systematically highlight the recent progress and future trend. Here, we first summarize the fundamental mechanism of light‐to‐heat and various categories of typical photothermal materials such as plasmonic metals, semiconductors, and carbon‐based materials. Then, we present the basic mechanism, design principle, and recent development of diversified solar‐driven water purification/harvesting protocols, including photothermal‐assisted water evaporation, photothermal‐assisted MD, photothermal‐assisted crude oil cleanup, photothermal‐enhanced photocatalysis for dye degradation, and photothermal‐assisted water harvesting from air (shown in **Figure** **1**a). Finally, conclusions and remaining challenges in this field will be discussed, hoping to provide a novel insight into harnessing solar energy to produce clean and safe drinking water, with great implications for fulfilling the blue dream of low‐energy water purification/harvesting through multidisciplinary research collaborations within solar‐driven photothermal effect and other advanced technologies.

## 2 Recent Development and Progress of Photothermal Materials

Photothermal materials, as the core component of light‐to‐heat convertor, play a very important role in utilizing solar energy. From the perspective of practical applications, the appealing photothermal materials should be equipped with some distinct features of strong and broadband light absorption, abundant and sustainable material sources, scalable manufacturing process, and low cost. Over the past few years, there has aroused a surge of research interest in exploring diversified state‐of‐the‐art photothermal materials to meet different requirements. In this section, according to different mechanisms of light‐to‐heat, we clarify photothermal materials into three categories, denoted as plasmonic‐based, semiconductor‐based, and carbon‐based solar absorbers (Figure 1b). Moreover, we highlight recent progress of carbon‐based absorbers, which have exhibited dominated potential in future practical application as next‐generation photothermal materials. The aim of this section is to offer a better understanding on the fundamental theory, and guide us to design and develop high‐performance photothermal materials to satisfy different demands of both basic research and practical application.

### 2.1 Plasmonic‐Based Solar Absorbers

As a common and unique optical phenomenon, the localized surface plasmon resonance effect of metal nanoparticles obeys the Drude–Lorentz model of solid‐state physics.^30^ When the incident light frequency can match the inherent oscillation frequency of free electrons within metal surfaces, it will trigger the generation of photoexcited hot electrons, followed by the oscillation with incident electromagnetic field. Then these photoexcited hot carriers tend to redistribute their energy via electron–electron scattering process, so as to raise the temperature of metal surface and its surrounding (i.e., plasmonic photothermal effect).^31^ On the basis of above interaction mechanism, Orrit and co‐workers first reported a plasmonic photothermal effect of nanometer‐sized gold particles in 2002.^32^ Benefiting from the achievements in the area of nanoscience and nanotechnology, there have been numerous kinds of metals used as plasmonic photothermal agents so far, such as gold (Au),^33^, ^34^, ^35^ silver (Ag),^36^ platinum,^37^ palladium,^38^ and aluminum.^39^ However, these plasmonic metals only exhibit high absorption efficiency for a few characterized wavelengths, resulting in low light‐to‐heat efficiency. Although the absorption wavelength region is able to be well tuned via adjusting the shape, size, and nanostructure of metals, the whole manufacturing process undergoes complex manipulation and relatively high cost. In addition, if designed into flexible and portable plasmonic photothermal devices, these plasmonic metals usually have to be compounded with some supporting substrates, including nanoporous anodic aluminum oxide (AAO),^40^ wood,^41^ porous membrane,^42^, ^43^ and hydrogel,^44^ through blending method or in situ growth.

### 2.2 Semiconductor‐Based Solar Absorbers

Black semiconductor materials have been frequently used as photothermal agents, and their heat generation mechanism is different from aforementioned plasmonic metals.^45^ First, when the energy of incident light is higher than or equal to the bandgap of semiconductor, the electron–hole pairs will be motivated and generated within semiconductor. Second, these photoexcited electrons and holes can return to the band state and then release the extra energy to improve the local temperature via nonradiative relaxation. However, most wide‐bandgap semiconductors exhibit relatively narrow absorption wavelength. For example, typical titanium dioxide (TiO_2_), with a bandgap of 3.2 eV, can only respond to UV region (<400 nm) and have almost no absorption for visible and near‐infrared regions.^46^ To extend its absorption wavelength, black TiO*_x_* nanoparticle was successfully constructed through magnesium (Mg) reduction of white P25 TiO_2_ nanocrystals to reduce its bandgap. Thanks to this treatment, its absorption wavelength is greatly broadened and is able to cover the whole solar spectrum, not only in UV regions but also in the visible and near‐infrared spectral regions.^47^ In addition, a series of new narrow‐bandgap semiconductors have been developed as the promising alternative to realize full‐spectrum solar‐to‐heat, including CuS,^48^, ^49^ Ti_2_O_3_,^45^ TiN,^50^ TiAlN,^51^ CuFeSe_2_,^52^ and Te.^53^ It should be noted that their absorption efficiency can only reach 90% or lower in spite of covering full‐spectrum solar. Recently, owing to the fast development of nanotechnology, some emerging 2D semiconductors materials, e.g., MoS_2_,^54^, ^55^ MXene,^56^ and black phosphorus,^57^, ^58^ are similarly utilized as photothermal materials because of their large surface area and strong light absorption ability. In 2017, Ti_3_C_2_ from the family of MXene was reported as a photothermal agent to harvest a perfect light‐to‐heat conversion efficiency of 100%. However, the major challenge lies in complicated and harsh fabrication processing conditions (i.e., high temperature and pressure) and poor stability toward air or water (especially black phosphorus). Additionally, these semiconductor nanomaterials are very difficult to be embedded into the supporting substrates using in situ growth method due to harsh reaction conditions.

### 2.3 Carbon‐Based Solar Absorbers

**Figure 2:**
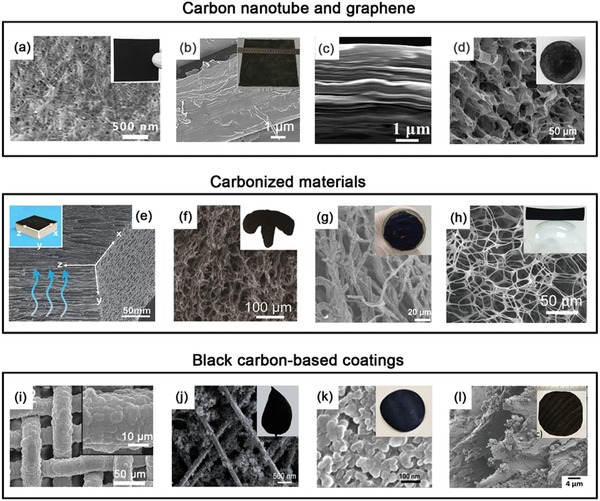
SEM images and digital pictures of diverse carbon‐based solar absorbers. a) CNTs film on the wood. Reproduced with permission.^60^ Copyright 2017, Wiley‐VCH. b) rGO coatings on the silk fabric. Reproduced with permission.^61^ Copyright 2018, Royal Society of Chemistry. c) rGO film via vacuum filtration. Reproduced with permission.^62^ Copyright 2017, American Chemical Society. d) rGO aerogel via freezing drying. Reproduced with permission.^63^ Copyright 2017, Wiley‐VCH. e) Carbonized wood. Reproduced with permission.^64^ Copyright 2018, Wiley‐VCH. f) Carbonized mushroom. Reproduced with permission.^65^ Copyright 2017, Wiley‐VCH. g) Carbonized cotton. Reproduced with permission.^66^ Copyright 2018, Royal Society of Chemistry. h) Carbonized foam. Reproduced with permission.^67^ Copyright 2018, Wiley‐VCH. i) PPy‐coated stainless‐steel mesh. Reproduced with permission.^68^ Copyright 2015, Wiley‐VCH. j) CB‐coated leaf. Reproduced with permission.^69^ Copyright 2018, Wiley‐VCH. k) Chinese ink‐modified membrane. Reproduced with permission.^70^ Copyright 2019, Wiley‐VCH. l) PDA‐coated wood. Reproduced with permission.^71^ Copyright 2017, Wiley‐VCH.

From amorphous carbon to graphite, carbon nanotubes (CNTs) and graphene, numerous carbon materials have been explored as the superior candidates to photothermal agents. Compared with plasmonic metals and semiconductors, photothermal carbon materials, especially some carbonized materials and black‐carbon‐based coatings, possess some unrivalled advantages: abundant sources, low cost, excellent processability, high thermal conductivity, and superior light absorption efficiency. Their heat generation mechanism is mainly through the thermal vibration of molecules. After absorbing incident solar light, carbon materials tend to be an excited state with high‐frequency phonon modes, and then turn to the low‐frequency modes via a phonon–phonon coupling process to release the energy, so as to generate heat energy.^59^ In this context, we emphasize the recent development of photothermal carbon materials divided into three categories: carbon nanotube and graphene, carbonized materials and black‐carbon‐based coatings (typical examples are shown in **Figure** **2**).

#### 2.3.1 Carbon Nanotube and Graphene

Different from conventional amorphous carbon, the molecular structure of CNTs and graphene has a large number of conjugated π bonds. These increased conjugated π bonds will result in a red‐shift of the absorption light spectrum, which greatly benefits in the solar energy utilization efficiency. As typical 1D nanomaterials, CNTs have two different kinds of configurations: single‐walled carbon nanotubes (SWCNTs) and multi‐walled carbon nanotubes (MWCNTs). Due to strong absorption property (up to 98% in visible and near‐infrared region)^60^ and high thermal conductivity (6000 W m^−1^ k^−1^),^72^ these CNT‐based photothermal convertors can not only efficiently generate a lot of thermal energy, but also fast transfer thermal energy into surrounding medium via radiation and conduction method. Up to date, two strategies are frequently used to prepare high‐performance CNT‐based photothermal devices: a) serving as the photothermal nanofillers^73^ and b) assembling into films,^60^, ^74^ or coatings.^75^ For example, Wang and co‐workers demonstrated that CNT‐coated sponges display 99% sunlight harvesting performance, and their surface equilibrium temperature can reach up to 88 °C,^75^ implying a remarkable photothermal conversion efficiency. However, the hydrophobicity nature of CNTs will bring some obstacles to their processability, and thus they usually require extra hydrophilic treatments.

Over the past decade, graphene has been considered as the most typical atom‐thick 2D material and has been widely applied in a wide range of material fields.^76^, ^77^, ^78^ Apart from the above‐mentioned advantages of CNTs as photothermal agent, the optical property and surface property (i.e., hydrophilicity and surface charge) of graphene are able to be elegantly tuned through regulating its oxidation degree (e.g., GO: graphene oxide or rGO: reduced graphene oxide). These graphene‐based derivatives could also be used as photothermal nanofillers, and a typical example was a composite photothermal hydrogel with a hydrophilic polymer framework (polyvinyl alcohol, PVA) and rGO as solar absorber.^79^ Thanks to the fast progress of micro–nano fabrication techniques, graphene‐based derivatives can be easily processed into various bulk materials (i.e., 2D film^80^, ^81^ and 3D aerogel^62^, ^63^, ^82^) as photothermal convertors. For instant, Hu and co‐workers used layer‐by‐layer 3D printing to prepare a GO‐based photothermal water evaporation device with an efficient broadband solar absorption (>97%), and its whole structure consisted of three parts: GO/CNTs layer, GO/nanofibrillated cellulose (NFC) layer and GO/NFC wall.^83^ Besides, a beam of laser could be also utilized to process the GO aerogel into highly vertically ordered pillar array of graphene‐assembled frameworks, and this unique structure is very useful for photothermal water evaporation.^84^ In spite of existing numerous graphene‐based photothermal convertors, the trade‐off between the optical property and the hydrophilicity of graphene‐based derivatives still remains a great challenge (i.e., rGO exhibits better absorption ability yet poorer hydrophilicity compared with GO).

#### 2.3.2 Carbonized Materials

Carbonization process has become a powerful and versatile strategy to synthesize carbon materials from organic biomass or biochar via a high‐temperature‐induced pyrolysis mechanism.^85^ Inspired by this technology, many researchers used some common, abundant, and low‐cost organic porous materials (e.g., foam,^67^, ^86^, ^87^, ^88^ lotus seedpods,^89^ wood,^64^ bamboo,^90^ and cotton^66^) to readily fabricate different photothermal convertors. Very recently, a natural mushroom was employed as the carbon precursor to produce a new‐fire solar steam‐generation devices. After carbonization treatment, the absorption efficiency of solar energy dramatically increases from 79% to 96%.^65^ Furthermore, such flexible choice of carbon precursors leaves more freedom on developing diversified photothermal convertors, tremendously broadening its development and applications. Unfortunately, the carbonization process usually suffers from a relatively high temperature (up to 500 °C), and thus it inevitably poses a great threat on the structure damage of inherent materials, including the shrinkage of volume and the reduction of pore size and porosity. Additionally, the mechanical strength of carbonization materials is also a problem worthy of careful consideration.

#### 2.3.3 Black‐Carbon‐Based Coatings

Surface modification technology has received immense interest and has the capacity of rendering the materials with multiple properties and functions, even without compromising their inherent structures (i.e., conformal function).^91^, ^92^ As expected, it can be also used to prepare black‐carbon‐based coatings on the substrates to improve their optical property. Recently, polypyrrole (PPy) has been widely employed as the photothermal coating due to its features of black color, low‐cost, and easy‐to‐fabricate. For instance, pyrrole can be facilely synthesized into PPy coatings on porous substrates like stainless‐steel mesh or poly(vinylidene fluoride) (PVDF) membrane via an oxidant‐induced polymerization,^93^ electropolymerization process,^68^ or chemical vapor deposition polymerization.^94^ Additionally, low‐cost carbon black (CB) nanoparticles and Chinese black ink are able to be uniformly deposited on the various substrates as a highly efficient solar absorber by a facile spray coating or dip‐coating approach.^69^, ^95^, ^96^ Although there are abundant modification protocols, the major concern of the abovementioned black carbon coatings is the poor adhesion to the substrates. As a result, Chinese black ink as the solar absorber coating was required to be further stabilized by atomic layer deposition (ALD) technique to improve the interfacial adhesion to the substrate and prevent the ink dispersing into the water.^70^

In 2007, mussel‐inspired chemistry, especially polydopamine (PDA), was first reported and has been turned into the promising potential method for surface modification because of the robust interfacial adhesion, facile reaction condition and versatile functionalization accessibility.^97^, ^98^, ^99^ It is well known that the structure of PDA is similar to eumelanin in organism, and thus it would enable PDA coating with excellent light absorption ability.^100^ Based on these properties, this PDA deposition technology was recently employed to readily fabricate black PDA coating on the wood surface as the photothermal convertor.^71^ Beyond above report, a series of PDA coatings have been developed as the photothermal agents for various applications, including photothermal therapy,^101^ photothermal antibacteria,^102^ photothermal‐assisted MD,^103^ photothermal‐assisted water evaporation,^104^, ^105^ and photothermal‐assisted crude oil cleanup.^106^ However, due to the limitation of its molecular structure, PDA coatings can only have high absorption efficiency within UV and visible regions, without covering the full solar spectrum.^71^ Given the feature of highly conjugated and delocalized molecule structure, a flexible porphyrin organic framework was recently exploited and in situ grew on a diverse range of porous substrates as the solar absorber, exhibiting a high absorption efficiency of 95% over the wavelength range from 300 to 1300 nm.^107^

## 3 Emerging Photothermal‐Assisted Water Purification/Harvesting Technologies

Owing to its unique self‐heating feature powered by solar energy, photothermal effect has been elegantly integrated into some conventional technologies to fulfill unrivalled performance applied in various environment‐ and energy‐related fields. Particularly, it has injected new vitality to realize the dream of low‐energy water purification/harvesting to reconcile the water–energy nexus. In this section, we emphasize the current cutting‐edge photothermal applications toward the freshwater crisis, such as photothermal‐assisted water evaporation, photothermal‐assisted MD, photothermal‐assisted crude oil cleanup, photothermal‐enhanced photocatalysis for dye degradation and photothermal‐assisted water harvesting from air, together with their advantages over conventional technologies.

### 3.1 Photothermal‐Assisted Water Evaporation

#### 3.1.1 Development Trend of Solar‐Driven Water Evaporator

As a ubiquitous and spontaneous phenomenon through a phase change process, water evaporation is of vital importance to the natural water cycle, which is significantly influenced by many factors such as temperature, atmospheric pressure and humidity. During this phase change process, liquid water is able to be straightway transformed into steam after adsorbing thermal energy, leaving behind salt and contaminant impurities. Therefore, it is expected to have the access to obtain freshwater from seawater or polluted water. However, this spontaneous process generally suffers from low evaporation efficiency, which is far away from the demand of large‐scale freshwater supplies. In order to facilitate water evaporation process, the conventional approach is to use extra energy, such as burning a large number of fossil fuels, to heat up the bulk water. Obviously, it is not a green and economic strategy because of secondary pollution and large energy consumption. To overcome this obstacle, many scientists have developed and utilized numerous reflecting mirrors as the solar collector devices to concentrate solar energy and warm up the bulk water, instead of conventional fossil fuels.^108^ Although this solar‐driven device exhibits certain ability of addressing the issue of energy consumption, it still undergoes some drawbacks as follows. The first one is that the use of numerous reflecting mirrors results in the increased investment cost to some extent, thus restricting its practical applications in some emergencies (e.g., offshore and remote area) or household use. The second one is that the water evaporation efficiency still tends to be unsatisfied due to a certain amount of light losses and low light‐to‐heat efficiency. Thus, it is highly desired for developing a low‐cost, portable, and high‐efficient solar‐driven steam generation device.

**Figure 3:**
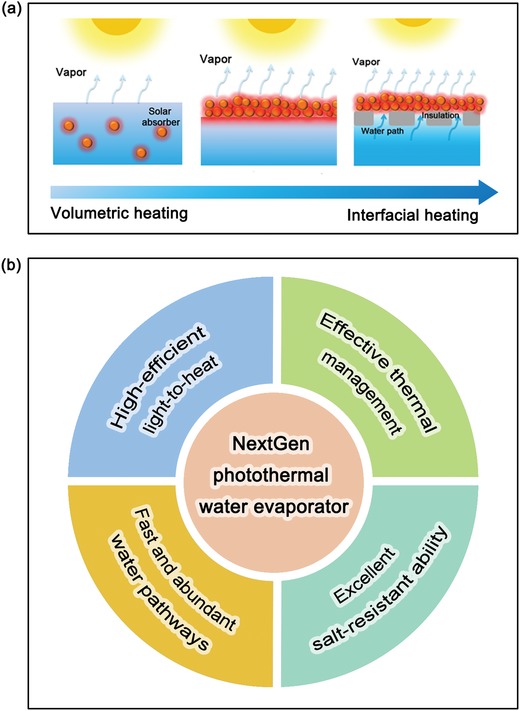
a) Schematic illustration of the development trend of solar steam generation device. b) Criteria of NextGen photothermal water evaporator.

Motivated by the opportunity offered by photothermal effect, intensive efforts have been recently paid to integrate diverse photothermal materials into solar steam generation devices to improve water evaporation efficiency. Compared with conventional solar‐driven steam generation, the introduction of photothermal materials will tremendously enhance the solar utilization efficiency to generate more thermal energy, which is very helpful to rapidly heat up water within just a few seconds to further induce high‐efficient water evaporation. According to the location of photothermal materials in the working fluid, there are two typical photothermal‐assisted solar steam generation modes, denoted as the volumetric heating system (nanofluid) and interfacial heating system shown in **Figure** **3**a. Among them, the volumetric heating system was earlier proposed and applied for water evaporation where photothermal materials were directly dispersed into the water. Upon illumination, the dispersed photothermal materials can absorb solar light and then convert it into heat to warm up the surrounding water for steam generation. Currently, there are two kinds of evaporation mechanisms: the emergence of nanobubbles^21^ and bulk heating.^109^ However, two critical factors greatly limit its practical applications. First, these dispersed photothermal materials were required to recycle from the working water after water evaporation process otherwise it would become secondary pollution. Thus it is impossible to apply in some open systems like rivers, oceans, and lakes. To solve this problem, black magnetic Fe_3_O_4_ microsphere was synthesized as the dispersed photothermal convertor for water evaporation, and its inherent magnetic property enabled effective recycle from the working fluid.^109^ However, unlike Fe_3_O_4_, most photothermal materials mentioned in Section 2 have no magnetic characteristic, and thus it urgently calls for new materials and approaches to solve this issue. Second, the surrounding water media of dispersed photothermal materials would reduce solar utilization to some extent because of light reflection and refraction, as well as inevitably increase heat losses to the surrounding water.^21^

Taken aforementioned issues of volumetric heating system into careful consideration, a new‐fire solar‐driven steam generation mode has been proposed and developed, namely interfacial heating system. Differently, photothermal materials are located and floated on the water surface (i.e., at the air–water interface) in the interfacial heating system. Under solar illumination, the floated photothermal materials are able to efficiently absorb the sunlight and convert it into heat to form a hot‐zone on the materials surface for heating up and evaporating the working liquid, and then as‐formed vapor is released and condensed into fresh water. This floating system is able to not only avoid the recycle procedure of photothermal materials, but also realize the localized heating effect to confine the as‐generated heat at the interface to reduce heat losses. Thanks to above improvements, interfacial heating system exhibits higher solar‐to‐vapor conversion efficiency than that of volumetric heating system. For instance, under one sun irradiation (1 kW m^−2^), the solar‐to‐vapor conversion efficiency of AuF suspensions is only 36%, which is much lower than that of AuF 2D film (≈52%) and 3D silica/AuF gel systems (≈85%).^44^ In general, the solar‐to‐vapor conversion efficiency (η) can be quantified by the following equations^110^

(1) $$\eta =\frac{m\times \left(H_{v}+H_{s}\right)}{Q}$$

where *m*, *Q*, *H*_v_, and *H*_s_ represent the mass flux of water evaporation, the incidence solar light intensity, the latent heat of water evaporation (i.e., phase‐change enthalpy), and the sensible heat of water, respectively. *H*_v_ is determined by the vaporization temperature of the water/air interface (2453 kJ kg^−1^ at 20 °C and 2265 kJ kg^−1^ at 100 °C), and *H*_s_ is dependent on the specific heat of water (*c*_water_ = 4.186 kJ kg^−1^ °C^−1^) and its temperature change. On the basis of these equations, decreasing the losses of as‐generated heat plays a critical role in the improvement of solar‐to‐vapor efficiency. In aim to minimize heat losses and boost conversion efficiency, an insulating and hydrophilic porous supporter has been integrated into the floating system to avoid the direct contact between photothermal materials and bulk water, as shown in Figure 3a, which has been regarded as the next generation solar‐driven water evaporation device.^110^

Over the past five years, great progress has been made in the interfacial heating system for water evaporation from the selection of photothermal materials to the configuration optimization of evaporator devices. Several previous reviews have briefly summarized the diverse solar absorber materials and device designs of steam generators,^13^, ^23^, ^26^, ^27^, ^28^, ^29^, ^111^ whereas we will mainly focus on the latest representative strategies for enhancing water evaporation efficiency in the following part. To date, there are four strategies to improve water evaporation productivity, which are first proposed as crucial criteria of NextGen photothermal water evaporator: 1) superior light utilization ability and light‐to‐heat efficiency, 2) effective thermal management, 3) appreciable water channels and 4) excellent salt‐resistant ability (shown in Figure 3b). Then, we highlight the emerging applications associated with water evaporation process, including desalination, environmental remediation (i.e., the removal of organic dye, heavy metal, and bacteria) and electricity generation.

#### 3.1.2 Strategies for Enhancing Water Evaporation Efficiency

*Superior Light Utilization Ability and Light‐to‐Heat Efficiency*: As a core component, the selection and design of photothermal materials play a very crucial role and even directly affect the overall evaporation efficiency. As mentioned in Section 2, there are diverse photothermal materials with excellent light absorption ability and light‐to‐heat efficiency as the potential candidates. Besides, three approaches have been usually employed to further boost the light‐to‐heat efficiency for meeting different demands: hybrid approach, configuration design, and substrate selection.

*Hybrid Approach*: Hybridization of photothermal materials has been demonstrated to be a powerful tool to harvest satisfied light‐to‐heat performance, because it can take full use of the optical advantage of different solar absorbers to maximize the optical property in both absorption range and intensity. For example, in view of composite photothermal membranes containing 2D rGO and 1D MWCNTs,^74^ its surface temperature can reach up to 78 °C upon one sun irradiation, which is 10 and 5 °C higher than that of pure rGO and MWCNTs membranes, respectively. As a result, rGO/MWCNTs composite membranes possess the best solar‐to‐vapor conversion efficiency. Similarly, GO/MWCNTs hybrid aerogel was prepared and exhibited more broadband and efficient solar absorption (around 92%) compared to the pure GO aerogel.^63^ Besides, when the plasmonic Au meets carbon‐based materials, their water steam productivity (water evaporation per unit mass of photothermal agents) is as high as 179.5 mg mg^−1^ after 30 min under irradiation of 0.51 W cm^−2^, which is much higher than that of the pure carbon spheres (120.8 mg mg^−1^) and Au nanoparticles (51.3 mg mg^−1^), respectively.^112^ It is attributed that the synergic effect of Au nanoparticles and carbon spheres leads to enhanced optical absorptions and reinforced hot electron–phonon interactions at the interface. In one word, this delicate synergistic effect can bestow photothermal hybridization materials with remarkably enhanced light‐to‐heat property.

**Figure 4:**
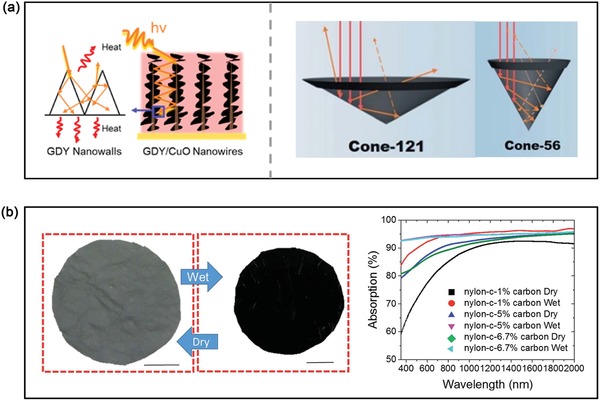
Advanced strategies to increase the light absorption ability of solar absorbers. a) Schematic illustration of the multiple reflection effects of incident light (left) and the reflection processes of incident light on different photothermal samples with suitable apex angle (right). left) Reproduced with permission.^113^ Copyright 2017, American Chemical Society. right) Reproduced with permission.^93^ Copyright 2018, Royal Society of Chemistry. b) Digital pictures and the corresponding UV–vis–NIR adsorption spectra of nylon‐C photothermal cloths in the dry and wet conditions. Reproduced with permission.^116^ Copyright 2018, Royal Society of Chemistry.

*Configuration Design and Substrate Selection*: Besides from improving the solar absorption, reducing the losses of incident light is another encouraging technology route to boost the photothermal property. When the incident light is irradiated into the surface of solar absorbers, the light reflection is usually accompanied with light absorption. And these reflected lights will return into the air and thus cannot be effectively utilized if the surface of solar absorber tends to be plane. Taken these into account, reducing or reusing the reflected lights is an effective approach to improve the solar utilization efficiency and water evaporation rate. Recently, researchers have found that configuration design and substrate selection of solar absorber are able to increase the times of light refection to realize multiple reflections or reduce the occurrence of reflected light. As a typical example of configuration design reported in 2017, a graphdiyne/CuO‐based multidimensional architecture could achieve 91% of photothermal efficiency and 1.55 kg m^−2^ h^−1^ of water evaporation rate under 1 kW m^−2^ illumination (**Figure** **4**a).^113^ Such high performance was ascribed to their unique configuration design where 2D graphdiyne nanosheets were fully decorated on the surface of vertical CuO nanowires to form the hierarchical architecture. Owing to this hierarchical architecture, the solar absorber has the ability of reusing reflected light via multiple reflections, resulting in the increased solar utilization efficiency by 8%. Subsequently, a bio‐inspired 3D photothermal cone with a tunable apex angle and base was reported to achieve superior solar‐driven evaporation (Figure 4a).^93^ With the reduction of apex angle and base, the diffuse reflectance of photothermal cone continually diminished due to increase the times of reflection. Similarly, a deployable, 3D solar evaporator was fabricated by folding and unfolding periodic pleats inspired from Miura‐ori tessellation, and this device could yield an extraordinary solar energy efficiency close to 100% upon one sun illumination.^114^ Most recently, Xu and co‐workers demonstrated that a copper–silicon (HCS) based membrane with marvelous hierarchical micro/nanostructures could exhibit highly efficient and broadband solar absorption ability at an arbitrary incident angle, ascribed to the light‐trapping effect, multiple light interactions and plasmonic enhancement effect.^115^ All these results indicate that the rationally designed 3D morphology has the great potential for minimizing light reflection to enhance the light‐to‐heat property.

The rational substrate selection of solar absorbers is another appealing route to realize highly efficient light‐to‐heat conversion. For example, a plasmon‐enhanced solar steam generation device was proposed and prepared by the self‐assembly of Au nanoparticles into a 3D nanoporous AAO substrate.^117^ In this case, on the one hand that the template effect of 3D porous AAO substrate can serve as an efficient light trapping media to strongly scatter the incident light through internal reflections, so as to extensively enhance solar absorption. On the other hand, 3D porous AAO can also effectively reduce the mismatched refractive index between solar absorber and air to decrease reflection losses. Generally, when light travels from one media to another, the reflectivity (*R*) at the interface can be briefly calculated according to the Fresnel equation^116^

(2) $$R={\left(\frac{n_{1}-n_{2}}{n_{1}+n_{2}}\right)}^{2}$$

where *n*_1_ and *n*_2_ are the refractive index for media 1 and media 2, respectively. Therefore, it is worth pointing out that it will completely eliminate the reflection losses if solar absorber possesses the same refractive index as surrounding media. Based on this theory, Wang and co‐workers recently adopted low refractive index of quartz glass (*n*_glass_ = 1.46, slightly higher than refractive index of water, *n*_water_ = 1.33) as the substrate to load CuCr_2_O_4_ nanoparticles, exhibiting excellent underwater black property and high water evaporation rate (1.319 kg m^−2^ h^−1^) under one sun.^118^ It is interesting that the optical property of CuCr_2_O_4_/quartz glass was dramatically better in wet state than that in dry state, attributed to the matched refractive index between substrate and water. Actually, this underwater black property is more relevant to solar‐driven water evaporation because the solar absorber is readily wetted by water or vapor to present wet state. Similarly, a durable, flexible, washable, and effective nonwoven photothermal cloth was successfully constructed using low refractive index of nylon 6 (*n* = 1.53) as the substrate, which could absorb over 94% of the solar light under wet state (Figure 4b).^116^ Therefore, the Fresnel equation is able to provide useful theoretical basis to guide us to screen the optimal photothermal substrates to obtain high‐performance water evaporation.

*Effective Thermal Management*: Although photothermal‐generated heat is confined and localized at the interface during the interfacial heating system, it is inevitable that the energy dissipation and losses will be accompanied with water evaporation process. In general, there are two adverse energy transfer processes to diminish the utilization efficiency of as‐generated heat: 1) conductive and radiative heat from the solar absorber to the underlying water, and 2) radiative and convective heat from the solar absorber to the surrounding. To eliminate these adverse effects, intensive efforts have been devoted to designing and developing effective thermal management strategies.

**Figure 5:**
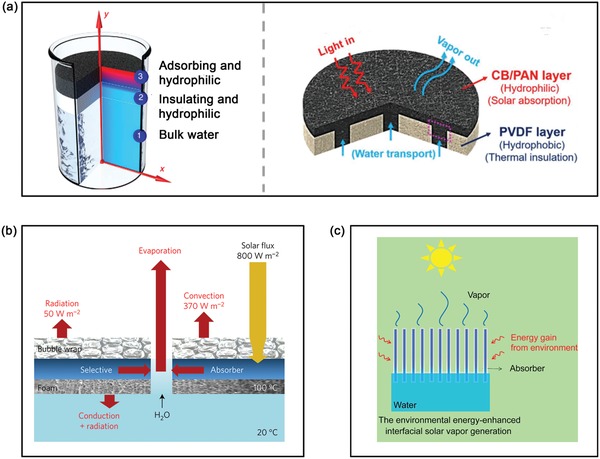
Various thermal management methods to reduce heat losses. a) A low‐thermal‐conductivity or insulating supporter between the solar absorbers and bulk water: carbon foam (left), and PVDF film (right). left) Reproduced with permission.^110^ Copyright 2014, Springer Nature. right) Reproduced with permission.^119^ Copyright 2019, Wiley‐VCH. b) Energy balance and heat transfer of solar evaporator with a bubble wrap under one sun. Reproduced with permission.^120^ Copyright 2016, Springer Nature. c) Schematics diagram of the environmental energy‐enhanced interfacial solar steam generation. Reproduced with permission.^121^ Copyright 2018, Elsevier.

*Reducing Heat Transfer Downward to the Underlying Water*: When solar absorbers are directly floated onto water surface, a part of heat transfer downward will heat up the entire body of water, which actually has no contribution to water evaporation. As the decrease of contact area between solar absorber and water, solar‐to‐vapor efficiency can increase from 73.5% to 93.8%.^93^ As a consequence, the effective and common solution is to reduce the contact area to minimize the conductive and radiative heat from the evaporation surface. In 2014, for the first time, Chen's group demonstrated a new floating two‐layer structure for steam generation (**Figure** **5**a), consisting of a carbon foam layer supporting an exfoliated graphite layer. In this special structure, the bottom carbon foam is thermally insulating to reduce heat losses to the underlying bulk water, and the top exfoliated graphite layer acts as the solar absorber. This two‐layer structure design is able to effectively avoid the direct contact between solar absorber and bulk water, bestowing steam device with a solar‐to‐vapor efficiency of 85% at 10 kW m^−2^ solar illumination.^110^ Since then, the introduction of the insulating porous supporters has been considered as the most effective method to reduce heat losses and realize thermal management. Until now, a series of low thermal conductivity materials have been exploited as the insulating supporter, including polyethylene membrane (0.448 W m^−1^ K^−1^),^48^ cotton (0.04 W m^−1^ K^−1^),^66^ wood (0.11–0.36 W m^−1^ K^−1^),^64^, ^122^ polystyrene foam (≈0.04 W m^−1^ K^−1^),^65^ and plant fiber sponges (0.103 W m^−1^ K^−1^),^123^ and electrospun polyvinylidene fluoride (PVDF) layers (Figure 5a).^119^ Among them, it should be pointed out that a reverse‐tree design was reported as a cost‐effective, scalable and high‐performance steam generation device because of much lower thermal conductivity (0.11 W m^−1^ K^−1^) than that of normal wood structure.^64^ Apart from above‐mentioned two‐layer structure, diverse 3D bulk solar absorbers with low thermal conductivity and moderate thickness can also act as the photothermal convertor, as well as prevent heat losses to the bulk water, such as synthetic polymer foam (0.057 W m^−1^ K^−1^),^124^ GO aerogel (0.047–0.035 W m^−1^ K^−1^),^63^ silica/Au gel,^44^ and polymer hydrogel.^79^

*Preventing Heat Transfer to the Surrounding*: Generally, the top surface temperature of solar absorbers is relatively higher than that of the surrounding, and thus there is no doubt that the as‐generated heat will loss to the surrounding via the radiative and convective method. Assuming a black solar absorber with *T* = 100 °C and the minimum temperature (*T*_∞_ = 20 °C) for boiling water at ambient conditions, we calculated that the radiative and convective heat losses to the ambient is up to 680 W m^−2^ and 800 W m^−2^ (convection heat transfer coefficient is 10 W m^−2^ K) upon the one sun illumination (1000 W m^−2^), respectively, according to the following equation^120^

(3) $$\alpha \;q_{\mathrm{solar}}=m_{e}h_{e}+\varepsilon \sigma \left(T_{s}^{4}-T_{\infty }^{4}\right)+h_{c}\left(T_{s}-T_{\infty }\right)$$

where α represents the solar absorbance, *q*_solar_ is the solar energy flux, *m*_e_ is the water evaporation rate, *h*_e_ is evaporation enthalpy of water, ε is the emissivity of the solar absorber surface, σ represents the Stefan–Boltzmann constant, *T*_s_ is the working temperature, *T*_∞_ is the ambient environment temperature, and *h*_c_ is the convective heat transfer coefficient. It is noteworthy that the sum of the two kinds of heat losses (1480 W m^−2^) has exceeded the incident solar flux (1000 W m^−2^). Therefore, huge radiative and convective heat losses pose formidable challenge in achieving high‐performance solar steam generation, especially under obvious temperature difference between solar absorber and surrounding. To alleviate these heat losses, there are two typical routes applied for solar steam generator. The first route is to decrease the temperature of solar absorber to narrow the temperature difference to the surrounding. When carbonized mushrooms were used as solar steam‐generation device, water evaporation rate was as high as 1.475 kg m^−2^ h^−1^. It was resulted from that the specific structure of mushroom could increase evaporation surface to lead to low surface temperature (38 °C under one sun) so as to reduce radiative heat.^65^ In order to further study the influence of evaporation surface, three kinds of evaporators, such as 2D direct contact evaporator, 2D indirect contact evaporator and 3D artificial umbrella‐like evaporator, were developed to systematically compare the correlation between the surface temperature (i.e., evaporation surface) and water evaporation rate.^125^ It has found that 3D artificial umbrella‐like evaporator displays a lowest surface temperature (32.7 °C) but highest solar vapor generation efficiency (85%) under one sun than that of two other evaporators (39.5 °C/49% for 2D direct contact evaporator, 43.0 °C/76% for 2D indirect contact evaporator). As we can see, although the radiative heat losses can be effectively suppressed by decreasing working temperature, their solar vapor generation efficiency is still low than 90%. It is because that low working temperature also deteriorates the water evaporation efficiency. Therefore, it is very crucial to optimize the working temperature of solar evaporator.

Another feasible route is to implant the protective layer to prevent the radiative and convective heat losses. In 2016, a transparent bubble jacket wrap was mounted on the top of solar absorber for minimizing convective heat losses without affecting the vapor release (Figure 5b).^120^ In addition, a cermet‐coated copper sheet was employed as solar absorber with high solar absorbance and low thermal emissivity, which could suppress the radiative heat to some extent. Thanks to these two unique designs, the solar evaporator was capable of maintaining a steam temperature of 100 °C under one sun irradiation, which has been never realized in previous studies. However, this suppression method of convective losses was at the expense of light transmission, because solar transmittance of the bubble wrap was only 80%. Additionally, a new thermal management method was proposed that carbon‐coated paper as solar absorber was placed within a square hole of low thermal conductive polystyrene foam.^126^ In this configuration, the side surface of carbon‐coated paper was fully covered by polystyrene foam, which was able to prevent the radiative heat losses from other open areas to the surrounding. Thus, water evaporation rate was 2.0 times greater than that of carbon‐coated paper without above configuration design.

*Harvesting Heat Energy from the Surrounding*: Besides from the aforementioned thermal management strategies through reducing heat losses to the surrounding, Zhu and co‐workers recently reported an environmental energy‐enhanced interfacial solar vapor generator by minimizing the energy losses from the top surface of solar absorber and maximizing the energy gain from the environment (the side surface of solar absorber) (Figure 5c).^121^ In this case, due to the special configuration of vertical cylinder solar absorber, the temperature of side surface was lower than that of top side surfaces and even environmental temperature, because most of solar energy was absorbed by the top surface. As a consequence, extra energy can be harvested from the environment via the radiative heat originated from temperature difference, instead of heat losses to the surrounding. Thus, this solar generator can obtain higher enhancement factor and yield an evaporation rate exceeding the theoretical value (100% solar‐to‐vapor energy transfer efficiency under 100 mW cm^2^ of solar illumination). Similarly, Gan and co‐workers proposed a new architecture of solar steam generator (carbon‐coated paper–air–foam structure floating on top of water) to harvest extra energy from the warmer environment, so as to realize near perfect efficiency (100%).^127^ To the best of our knowledge, the above two cases are the first time to realize a net energy gain (i.e., low‐grade heat) from the environment during solar steam generation, with great implication for the development of high‐performance solar steam generation. Most recently, the introduction of electro‐thermal effect was also used to further enhance water evaporation productivity through as‐generated electricity of solar cell (i.e., energy gain from solar through light‐to‐electricity‐to‐heat). Due to the introduction of photo‐electro‐thermal effect of graphene‐based materials, water evaporation rate was around 2.01–2.61 kg m^−2^ h^−1^ upon solar illumination of 1 kW m^−2^ even without configuration optimization,^128^ which was much higher than that of previously reported graphene‐based evaporators.^61^, ^63^, ^81^, ^84^ Surprisingly, the clean water productivity of this evaporator arrays with only several square meters could reach up to 8.6 kg m^−2^, which could meet the daily water supply enough for tens of people.

**Figure 6:**
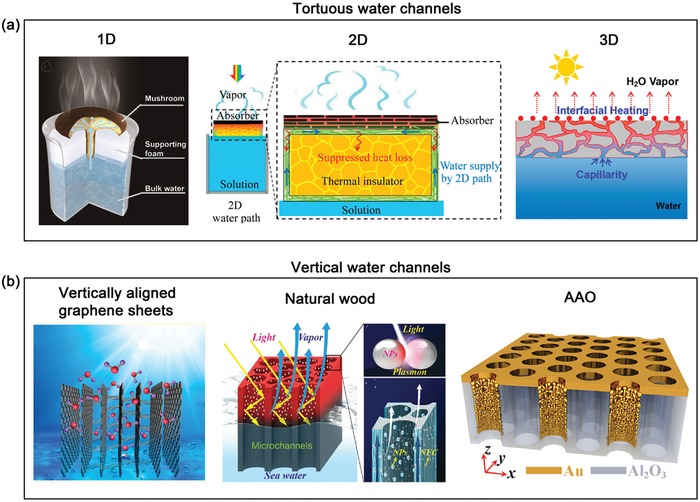
Various water channels of solar steam generator to increase the water supply. a) Diagram of a mushroom stipe as 1D water supply, GO film as 2D water supply, and porous PE/CuS membrane as 3D water supply. left) Reproduced with permission.^65^ Copyright 2017, Wiley‐VCH. middle) Reproduced with permission.^80^ Copyright 2016, National Academy of Sciences. right) Reproduced with permission.^48^ Copyright 2016, American Chemical Society. b) Vertical water channel in the vertically aligned graphene sheets membrane, wood, and nanoporous AAO. left) Reproduced with permission.^62^ Copyright 2017, American Chemical Society. middle) Reproduced with permission.^41^ Copyright 2018, Wiley‐VCH. right) Reproduced with permission.^117^ Copyright 2016, American Association for the Advancement of Science.

*Appreciable Water Channels*: Previous studies have found that interfacial heating system with an insulating supporter exhibited better solar‐to‐vapor property compared to the direct contact with water. Due to its indirect contact character between bulk water and solar absorber, continuous water supply plays a vital role in ensuring stable water evaporation efficiency. In nature, the transpiration process is very necessary for the metabolism and photosynthesis of plants, together with interfacial evaporation of water on the leaves. The evaporation on plant leaves can create a negative water vapor pressure, as well as capillary action within the numerous hydrophilic cellulose‐based channels of the plants, which have been regarded as the driving forces to trigger the mass transport (i.e., water and mineral substance) from the underground to the leaf through xylem vessels and lumina. Inspired by this principle, a large number of hydrophilic porous materials have been explored as appreciable capillary channels to draw mass transport to the surface of solar absorber for evaporation. Moreover, recent study has found that water transport channels could be turned on/off through the reversible transformation of microstructures to further tailor water evaporation rate, indicating the importance of water channels.^129^ According to the feature of channels, there are two main water channels applied for water supplies during solar steam devices: tortuous water channels and vertical water channels (**Figure** **6**).

**Figure 7:**
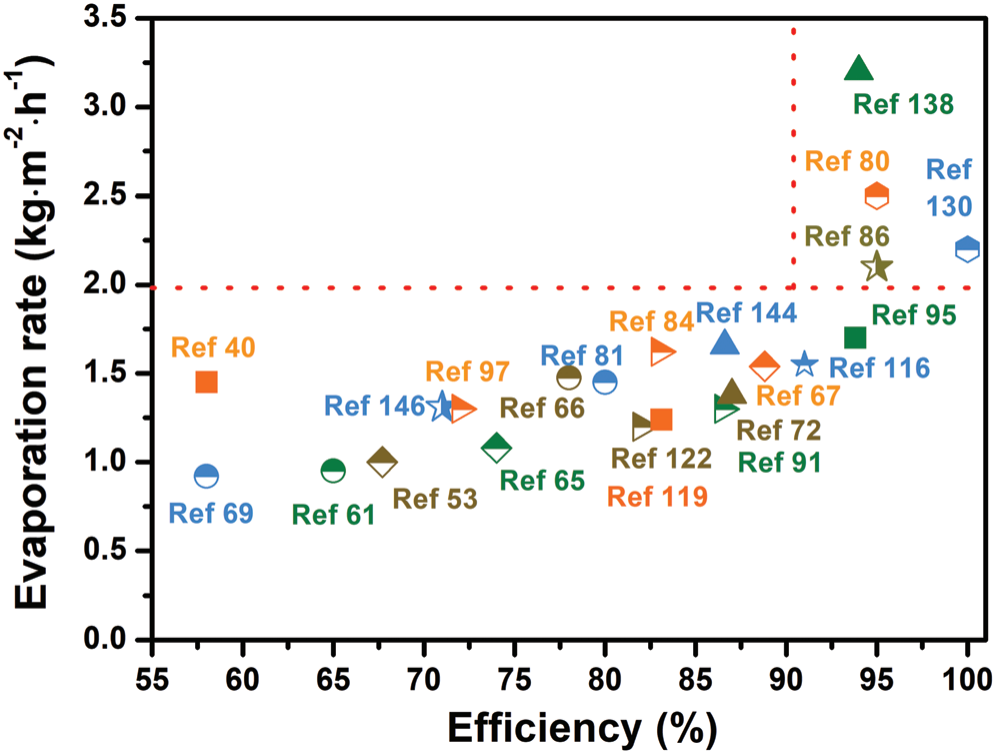
Water evaporation rate and efficiency of various previous reports. The evaporation experiments were conducted under the solar illumination of 1 kW m^−2^ and ambient temperature.

*Tortuous Water Channels*: In general, most materials possess the random structures, and thus tortuous water channel is commonly used for evaporation, which can be classified into three categories: 1D, 2D, and 3D water pathway. The typical example of 1D water pathway is natural mushroom stipe, and its hydrophilic fibrous stipe can pump up water into the mushroom by capillary force and confine the water path in a quasi‐1.^65^ Similarly, a cotton rod was designed as the stem of umbrella‐like solar evaporator to act as 1D water supply channel.^125^ The above 1D water pathway is able to not only provide enough water for evaporation, but also minimize heat conduction losses because of obviously reduced contact area between solar absorber and water. Besides, a confined 2D water pathway was designed for water supplies of solar absorber (laminated GO film) through a thin cellulose layer wrapped over the surface of a thermal insulator in 2016.^80^ In this confined 2D water pathway system, rapid water transport and reduced heat losses could be achieved at the same time, enabling the evaporator with 80% of solar‐to‐vapor efficiency under one sun illumination. But low porosity of this laminated GO film would significantly limit the amount of water transport to evaporation surface and release rate of vapor. Compared with 1D and 2D water pathway materials, 3D porous materials exhibit high porosity and hierarchically micro/nanoporous structures and thus can form strong capillary action, which is a much better choice for water supplies. Until now, numerous 3D porous materials have been applied to efficiently pump up water into solar absorber, including cellulose fabric,^3^ lotus seedpods,^89^ hydrophilic poly(tetra‐fluoroethylene) (PTFE) membrane,^130^ cotton,^66^ aerogels,^131^ sponge,^132^ and hydrogel.^79^, ^133^, ^134^, ^135^ It is worth noting that the polymer hydrogel‐based solar evaporator reported by Yu's group can harness 94% solar energy and exhibit a record high evaporated water rate of 3.2 kg m^−2^ h^−1^ under one sun irradiation, enough to satisfy daily individual drinking demands.^134^ In this PVA/PPy hydrogel, its 3D hierarchical network, consisting of internal gaps, micrometer channels and molecular meshes, has superior capacity of providing abundant water pathways to enable adequate and uniform water supplies to the evaporation surface through arterial pumping and branched diffusion triggered by capillary forces. Apart from capillary forces, hydrophilic polymer hydrogels usually possess increased osmotic pressure, which can also contribute water transport via osmotic swelling effects,^79^ similar to the role of hydrogel‐based draw agent during forward osmosis.^136^, ^137^ All these reasons endow this hydrogel‐based solar evaporator with the highest evaporation rate at present upon one sun irradiation (1 kW m^−2^), compared with previous reports as shown in **Figure** **7**. Obviously, it is very urgent to further improve water evaporator productivity despite the great level of effort in the development of solar steam generator, because most evaporator rates tend to be much lower than 2.0 kg m^−2^ h^−1^ under one sun.

*Vertical Water Channels*: Besides from the tortuous water channels, the vertical water channels have been also widely used in the water evaporators. Theoretically, for the same thickness and channel size, the vertical channels possess much higher water transport rate than the tortuous channels, due to their shorter transport pathway and lower transport barrier. Thus, designing vertical channels has the potential to effectively facilitate the capillary supply of water for evaporation and the preferential detachment of evaporated vapor simultaneously, thereby enhancing the overall water evaporation efficiency. Actually, natural woods contain numerous longitudinal cells and thin walled cells located in the radial direction, and such vertical nanochannel structure is significantly beneficial for them to pump up water and mineral substance from the soil to plants during the transpiration process. Thus, natural woods have been frequently used as the supporting of solar absorbers to utilize their vertical channels as water transport pathway.^41^, ^60^, ^70^, ^71^, ^138^ Thanks to this unique structure, wood‐based evaporator exhibited a higher water evaporation rate (1.31 kg m^−2^ h^−1^) than that of porous membrane‐based evaporator (0.75 kg m^−2^ h^−1^) under simulated 1 sun illumination.^70^ Apart from natural wood, a series of analogous materials with vertical channels have been fabricated for water evaporation owing to the fast development of nanomaterials preparation and processing technology, such as AAO,^39^, ^117^ vertically aligned carbon nanotube (VACNT) arrays,^139^ and vertically aligned graphene sheets membrane (VA‐GSM).^62^ For example, when a layer of VACNT arrays was used as solar steam generation, and its water evaporation rate was ten times higher than that of bare water under one sun illumination.^139^ It is mainly attributed to direct water transport pathways of vertical nanotube, and low friction and weak interfacial force between CNT and water, so as to trigger fast water transport for enhanced solar steam generation. However, it should be noted that high cost of this VACNT arrays greatly retards its practically large‐scale applications. Very recently, Qu and co‐workers adopted antifreeze‐assisted freezing technique to utilize the ice as the template to prepare the VA‐GSM for water evaporation.^62^ To emphasize the role of vertically run‐through channels, the evaporation performance of VA‐GSM was in‐depth compared with both rGO film and structure‐disordered rGO foam. Thanks to the profitable open channels for water transport and vapor release, VA‐GSM exhibits the highest water evaporation rate of 1.57 kg m^−2^ h^−1^ than that of other two materials upon one sun irradiation. Thus, the rational design and optimization of water transport channels have exhibited appealing potential to enhance the efficiency of solar steam generation.

*Excellent Salt‐Resistant Ability*: From the perspective of practical use, the reusability, durability, and recyclability of solar evaporator is also of very significance. During practical seawater evaporation, a large number of salts will be left behind and continuously accumulate on hydrophilic solar absorbers surface after water evaporation. Consequently, as‐aggregated salt crystals would not only weaken the optical property of hydrophilic solar absorbers, but also impede inherent capillary action to affect the water pumping pressure. Such an inevitable phenomenon is even likely to destroy the performance of water evaporation especially long‐term running under high salinity solutions. As shown in previous report, the CB/polyacrylonitrile (PAN) membrane displayed a drop in the evaporation rate by about 15% after 16 days running, and its light absorbance also decreased from 97% to 62% due to the salts accumulation.^95^ Hence, the issue of salts accumulation on the solar absorber surface is another bottleneck for the development of solar steam generator. To date, this hotspot has attracted increasing attention and motivated many researchers to exploit various strategies to address this issue and achieve salt‐resistant property, mainly including hydrophobic solar absorbers, specially designed hydrophilic solar absorbers, and device configuration design.

**Figure 8:**
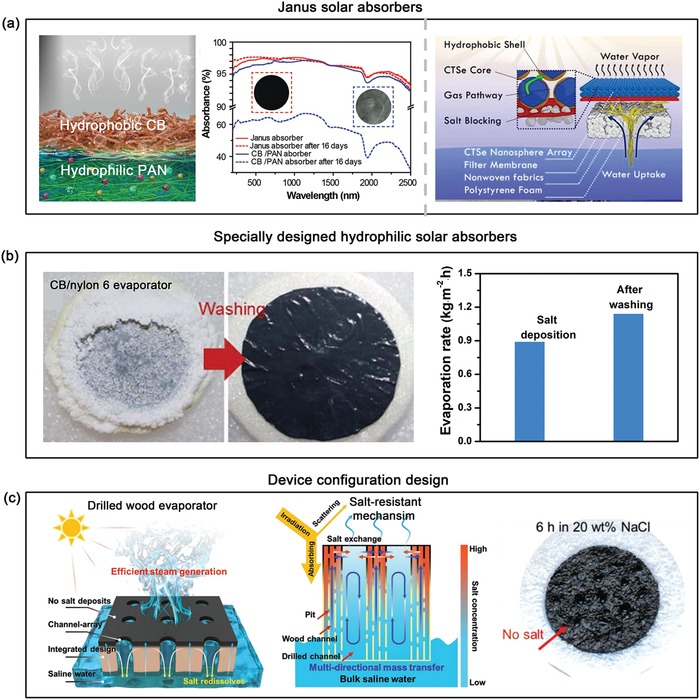
Various strategies for achieving salt‐resistant ability. a) Structures illustration of Janus absorber (CB/PMMA‐PAN) and its optical property (left), and hydrophilic/hydrophobic double layers solar steam devices (right) with salt blocking ability. left) Reproduced with permission.^95^ Copyright 2018, Wiley‐VCH. right) Reproduced with permission.^140^ Copyright 2018, Royal Society of Chemistry. b) Digital pictures of water evaporator before and after washing. Reproduced with permission.^116^ Copyright 2018, Royal Society of Chemistry. c) Schematic illustration of the self‐regenerating wood‐based solar evaporator, its salt‐resistant mechanism and the digital image. Reproduced with permission.^141^ Copyright 2019, Wiley‐VCH.

*Hydrophobic Solar Absorbers*: It has been well known that hydrophobic surface usually possesses nonwetting feature, and thus it is able to offer an effective route for preventing brine infiltration. On the basis of this principle, hydrophobic solar absorbers can realize the goal of salt‐resistant through avoiding the contact between solar absorber and brine. In 2018, for the first time, a Janus structure evaporator was proposed for solar steam generation consisting of two functional layers: upper hydrophobic CB‐coated polymethylmethacrylate (PMMA) layer as the solar absorber to harness solar as well as robust protector to resist salts accumulation, and bottom hydrophilic PAN layer as water storage to offer enough water supplies to the evaporation (**Figure** **8**a).^95^ As expected, the evaporation rate of janus evaporator was almost invariable after 45 min running, indicating excellent salt‐resistant ability, whereas the evaporation rate of conventional evaporator (i.e., hydrophilic solar absorber) slowed down by 15% because of obvious salts accumulation on the surface observed in SEM images. In spite of the success of above Janus evaporator, most solar absorbers are usually hydrophilic and thus hydrophobic modification approaches are required to be implanted. In order to minimize the adverse effects on evaporation, hydrophobic modified layers or technologies need to obey some features as follows: 1) enough transparent hydrophobic layers to enable intrinsic optical property without any losses, 2) conformal function of hydrophobic layers to prevent the clogging of the inherent channels, ensuring adequate water supplies and fast release of vapors. The typical example is that the light absorption materials such as Cu_2_SnSe_3_ (CTSe) and Cu_2_ZnSnSe_4_ (CZTSe) nanoparticles were first covered by hydrophobic oleylamine, and then as‐modified hydrophobic nanoparticles were filtrated on the hydrophilic porous membrane, namely hydrophilic/hydrophobic nanoporous double layer (HHNDL) solar steam generator like above‐mentioned Janus structure (Figure 8a).^140^ The results demonstrated that the evaporation rate of the HHNDL structure tends to be stable over 15 day continuous desalination without decay, and there is no salts accumulation observed onto the surface because of the salt blocking of hydrophobic CTSe layer. As a sharp contrast, the surface of hydrophilic CTSe layers has accumulated numerous salt crystals ranging from over nanometers to micrometers after only 10 h continuous desalination. In addition, hydrophobic trimethoxy(1*H*,1*H*,2*H*,2*H*‐perfluorodecyl)silane (PFDTMS)^142^ and poly(3,4‐ethylenedioxythiophene)‐poly(styrenesulfonate) (PEDOT‐PSS)^143^ were also used to modify d‐Ti_3_C_2_ nanosheets and carbon materials to prepare hydrophobic solar absorbers, respectively, endowing the water evaporator with excellent salt‐resistant ability applied in long‐term solar desalination. In one word, hydrophilic/hydrophobic janus solar steam generator exhibits superior capacity of solving the issue of salts accumulation, which is the most facile and effective strategy until now. Thus, it is very crucial to develop a facile, universal, and low‐cost modification route to construct the transparent and hydrophobic layers onto the surface of solar absorber.

*Specially Designed Hydrophilic Solar Absorbers*: Different from the aforementioned hydrophobic solar absorbers, some specially designed hydrophilic ones are similarly able to realize excellent salt‐resistant property, yet only two examples reported to date. For example, Wang and co‐workers demonstrated that a novel nonwoven photothermal cloth was fabricated by embedded CB nanoparticles into hydrophilic nylon nanofiber, exhibiting excellent antifouling ability.^116^ It is found that salt‐fouled nylon‐CB cloth can easily clean off the deposited salts via mild hand washed (Figure 8b), and as‐washed nylon‐CB cloth will fast recover its original evaporation rate. Very recently, a PVA/rGO hydrogels evaporator was reported and displayed tough crystalline antifouling functionality under a continuous solar desalination for 96 h, while various salt crystals appeared at the surface of rGO‐based evaporator.^79^ As a result, the implantation of hydrophilic PVA hydrogel renders the evaporator to promising antifouling performance for long‐term operation in a practical environment. Although these hydrophilic solar absorbers have exhibited certain salt‐resistant ability, the salt‐resistant mechanism behind still remains elusive. Therefore, it is highly desired to build the fundamental theory to guide us to design and screen this kind of hydrophilic solar absorbers.

*Device Configuration Design*: Optimizing device configuration design is also utilized to avoid the salts accumulation on the surface of solar absorbers. The typical example presented by Chen and co‐workers, an evaporator with simultaneous salt rejection and heat localization ability mainly contained a black fabric for solar absorption, a white fabric wick and polystyrene foam insulation. Thanks to their porous and hydrophilic feature, white cellulose fabric could fast pump up water to the solar‐absorbing evaporation structure, as well as effectively reject excess salts back to the underlying water.^3^ The according salt‐rejection mechanism is probably ascribed to advection and diffusion of concentrated salt down back into the body of water, and the driving force is resulted from the increased osmotic pressure within the evaporation structure where the salt concentration exceeds the ambient ocean concentration. Similarly, a self‐driven salt‐resistant mechanism was proposed to realize highly efficient and salt‐free solar steam generator through the free exchange of concentrated and dilute solutions during the evaporation process.^144^ Very recently, the natural wood with millimeter‐sized drilled channels and balsa wood with bimodal porous structure have been successfully demonstrated to possess excellent salt‐resistant ability.^141^, ^145^ This rationally designed channel‐array in natural wood can form salt concentration gradients between millimeter‐sized drilled channels (low salt concentration) and the microsized natural wood channels (high salt concentration) during evaporation process, allowing spontaneous interchannel salt exchange with the bulk solution to reach salt‐resistant effect (Figure 8c).^141^ As a result, this evaporator can work even at an extremely high salt concentration of 20 wt% without losing water evaporation rate at least 100 h.

#### 3.1.3 Applications Associated with Water Evaporation

With the great advancements made in the design, exploitation and optimization of photothermal materials and devices, solar steam generators have exhibited appealing potential to be applied in our actual production and life. As briefly above‐mentioned, solar steam generator has the ability of producing clean and drinkable water (collected distillate) leaving behind the inorganic salts (i.e., Na^+^, K^+^, Mg^2+^, Ca^2+^ Cu^2+^, Cr^3+^, Pb^2+^, and so on) and organic contaminants (i.e., dye). On the basis of this mechanism, solar steam generator can be applied for seawater desalination and wastewater treatment to harvest freshwater and realize environment remediation simultaneously. Additionally, this technology can be also employed to tune the plant transpiration through rationally engineering the absorber‐leaf interfaces,^69^ which is greatly beneficial to improve relative humidity and reduce temperature increment of surrounding air of the plant. Moreover, this solar‐driven evaporation process is also able to be elegantly combined with some other advanced technologies to generate high value‐added applications toward power generation, including salinity gradient induced power generation,^146^ pyroelectric/piezoelectric effects,^67^ thermoelectric module,^147^ and triboelectric generation.^44^ Thus, in this section, we first introduce the general applications of water evaporation such as seawater desalination and wastewater treatment, and then highlight the imminent chances of water evaporation‐assisted electricity generation.

**Figure 9:**
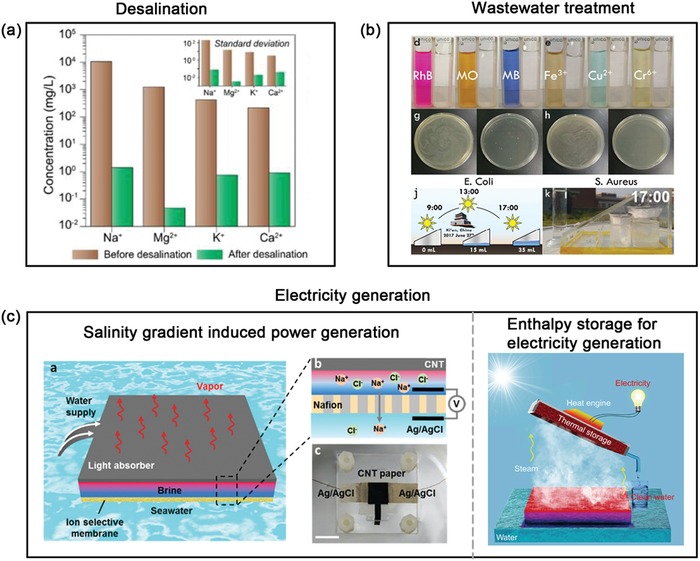
Solar‐driven water evaporation for different applications. a) Concentrations of four primary ions in the seawater before and after desalination under one sun using GO/CNT‐modified cellulose membrane as the solar absorber. Reproduced with permission.^114^ Copyright 2018, American Chemical Society. b) Purification effect of solar steam generator for different dyes, heavy metal ions, and bacteria. Reproduced with permission.^140^ Copyright 2018, Royal Society of Chemistry. c) Schematic of the salinity gradient induced power generation (left) and storage/recycling of the enthalpy for clean water and electricity (right) during solar‐driven photothermal desalination. left) Reproduced with permission.^146^ Copyright 2017, Royal Society of Chemistry. right) Reproduced with permission.^147^ Copyright 2018, Elsevier.

*Desalination*: Desalination technologies of membrane separation protocols have been considered as the most effective and common method to obtain clean and drinkable water from real seawater or other high salinity solutions.^18^, ^148^, ^149^ Recently, solar steam generation has also been considered as the appealing alternative to seawater desalination. Compared with conventional desalination technologies, its greatest advantage is to use sustainable and pollution‐free energy source for realizing low‐energy desalination. In addition, their features of easy‐to‐integrate, portability, and low‐cost/energy make it ideal to apply in open system like seas and rivers or remote off‐grid areas. For instance, the concentrations of four primary ions of Na^+^, Mg^2+^, K^+^, and Ca^2+^ in the seawater were significantly reduced by orders to 1.41, 0.05, 0.76, and 0.91 mg L^−1^ in the as‐collected distillate (**Figure** **9**a), respectively, which was far below drinking water salinity levels defined by World Health Organization (WHO) and US Environmental Protection Agency (EPA) Standard.^114^ Besides, the salinity of three artificial seawater like the Baltic sea (0.8 wt%), world ocean (3.5 wt%) and Dead Sea (10 wt%) after solar evaporation desalination was significantly decreased about four orders of magnitude.^79^ Recently, some reports have showed that the rejection of salt ions was up to 99.5% even under high original salt concentration (100 mg mL^−1^),^62^, ^140^ which was even higher than that of various conventional desalination technologies.^150^, ^151^, ^152^ Generally, high salinity would cause severe salt polarization phenomenon to greatly deteriorate the desalination performance, especially in those membrane‐based desalination technologies,^153^ while this solar‐driven desalination can effectively avoid this obstacle.

In order to fulfill the goal of zero liquid discharge (ZLD), the desalination process requires an effective residuals management method to recycle the byproduct of solid salts without compromising the production of freshwater. From the perspective of ZLD, the solar evaporator should have the ability to achieve efficient and continuous steam generation, as well as harvest salt crystals to fulfill the goal of perfect residuals management. However, most of the solar evaporators are very difficult to overcome the trade‐off between steam generation and salt crystallization (i.e., the salts accumulation on the evaporation surface significantly deteriorates the water evaporation performance). Recently, a 3D cup shaped solar evaporator was designed to rationally separate the solar absorber surface from the salt precipitation surface.^154^ Thanks to this specific configuration design, the salt crystals growth and precipitation was very significant on the outer cup wall, rather than the inner cup wall. As a results, this 3D cup evaporator could reach the simultaneous production of salt crystals and freshwater in over 120 h of nonstop operation. Additionally, Zhang and co‐workers first reported a solar steam generator with ZLD feature through precisely regulating the transport and distribution of salt solution in the photothermal material.^155^ It was very interesting that the salt crystallization appeared only at the edge of the evaporator due to a radial concentration gradient of salt from the center to the edge, spatially isolated from water evaporation surface. And these salts could fall off automatically under the assistance of gravity for effective residuals management. All these results imparted solar evaporator with superior ability of continuous solar steam generation and salt harvesting. Therefore, solar‐driven desalination technology shows tremendous potential to become a popular solution for sustainable seawater desalination and zero liquid discharge.

*Wastewater Treatment*: Besides from seawater desalination, solar steam generation can be also used to clean up heavy metal ions pollution, and printing and dyeing wastewater for environmental remediation. For instance, the concentration of heavy metal ions (e.g., Cu^2+^, Cr^3+^, and Pb^2+^) in the as‐collected distillate was 0.066, <0.01 mg L^−1^, and <0.01 mg L^−1^, respectively, which were much lower than that of effluent discharge content (the stipulated upper concentrations of Cu^2+^, Cr^3+^, and Pb^2+^ are 0.2, 0.5, and 0.2 mg L^−1^, respectively).^121^ The Hg^2+^ concentration levels were also reduced from 200 to 1 ppb to reach the EPA standard during water evaporation process, ascribed to the strong adsorption ability of MoS_2_ in the solar absorber.^156^ Some typical dyes such as Rhodamine B (RhB), methyl orange (MO) and methyl blue (MB) could achieve a rejection close to 100% (Figure 9b).^140^ Additionally, Escherichia coli and Staphylococcus aureus with ultrahigh number of 10^6^ can also be completely rejected during water evaporation process, and this solute blocking function can bestow water evaporation rate with stable value even under long‐term operation. As a contrast, these bacteria need to be removed before wastewater treatment during membrane technologies, because it is very easy to block membrane pores to result in the decreased performance. Most recently, the interfacial solar steam generation was first employed for effective off‐grid sterilization technique (≈99.99% inactivation of pathogen) using low cost and widely available biochar as solar absorber.^157^ All these results imply that solar steam generation has the ability to act as an eco‐friendly and low‐energy method to well remove contaminants in wastewater. However, the key concern of this method is the adsorption of dye and heavy metal ions on the solar absorbers surface, similar to aforementioned salts accumulation discussed in Section 3.1.2. And the dyes adsorption phenomenon will be significantly enhanced under the assistance of upward vapor flow during water evaporation.^158^ To solve this problem, the introduction of photocatalysts (in Section 3.4) and Janus evaporator have been considered as an effective solution. Besides from the organic dyes removal, Zhu and co‐workers first reported an asymmetric plasmonic solar absorber with dual functions for water purification and pollution detection.^159^ Interestingly, a minimal detection concentration down to ≈5 × 10^−12^ m and a Raman enhancement factor of ≈10^9^ at 10^−11^ m can be achieved when applied for chemical detection by surface enhanced Raman scattering (SERS).

*Electricity Generation*: During water evaporation, the salinity of water within the solar absorbers is relatively higher than that of bulk water because of evaporation‐induced salt accumulation or concentration, thus it would spontaneously form a salinity gradient. Given this phenomenon, Zhou and co‐workers first utilized the evaporation induced salinity gradient to generate electricity with a power value of 12.5 W m^2^ during steam production under one sun (Figure 9c).^146^ In this new concept of hybrid technology, a commercial Nafion membrane was located between the surface water and bulk seawater as the ion selective membrane to allow specific ions transport to form ions flow, so as to harvest electricity. Besides from the utilization of desalination process, as‐generated vapor and its condensation process were also conducted to produce electricity. For example, the dynamic mechanical and temperature fluctuations of as‐generated vapor were employed to successfully achieve waste energy‐to‐electricity conversion via the introduction of a ferroelectric fluoropolymer PVDF film to couple the pyroelectric and piezoelectric effects.^67^ In addition, a thermoelectric module could be used to reserve and recycle the thermal energy of vapor condensation during steam condensation to further obtain electricity with solar energy as the only energy input (Figure 9c).^147^ The open‐circuit voltage and short‐circuit current could reach to 4.15 V and 0.61 A, respectively, which was enough to enable continuous work of an electric fan (1 W) and 28 light‐emitting diodes (total power 1.5 W). However, this case requires a high energy input (up to 30 kW m^−2^). Recently, an effective approach to optimizing the configuration of thermoelectric module in the solar generator was elegantly demonstrated, which has the ability to harvest relatively good electricity output under one sun (1 kW m^−2^).^160^, ^161^ It is worth noting that the maximum output power was achieved to 0.05 mW (external resistance is about 2 Ω) when the thermoelectric module was used as heat insulator of water evaporator.^161^ Lastly but not least, Ho and co‐workers presented the triboelectric nanogenerator (TENG) devices could be well integrated into water evaporation system to effectively harness triboelectric energy generated from the flow of condensate vapor on a PTFE surface, and the maximum peak power obtained was 0.63 µW.^44^ Actually, these aforementioned electricity generation strategies are highly dependent on the state of as‐formed vapor, and thus the primary principle is to obtain robust and steady vapor without fluctuation during evaporation process. Although the power of above as‐generated electricity is far away from practical application, these proof‐of‐concepts provide an innovative approach for simultaneously obtaining fresh water and high value‐added electricity only using solar as the energy input, with great implication of solving freshwater and energy scarcity.

### 3.2 Photothermal‐Assisted Membrane Distillation

MD is a thermally driven separation technology through combining the membrane and distillation process, and has received continuous attention in both fundamental research and practical application.^162^, ^163^, ^164^ During typical MD configuration, a porous hydrophobic membrane acts as the core component to effectively separate hot feed and cold distillate. Due to the vapor‐pressure gradient on both sides of porous hydrophobic membrane, as‐evaporated vapor on the hot feed water side can diffuse across the membrane and then condense on the opposite side of the membrane to generate distillate. According to different condensation methods, MD can be clarified into four categories: direct contact membrane distillation (DCMD), vacuum membrane distillation (VMD), air gap membrane distillation (AGMD), and sweep gas membrane distillation (SGMD). Thanks to the hydrophobic feature of porous membranes, mass transfer only takes place in vapor phase, whereas the water or the solutes (i.e., macromolecules, colloids, and ions) in water can be completely rejected and stayed in the hot feed water. Surprisingly, MD is able to treat high‐salinity brines without the occurrence of concentration polarization, and its salinity treatment limit is higher than that of RO (around 80 g kg^−1^).^162^ Additionally, MD possesses much lower operating pressure than that of RO technology, which benefits in decreasing the formation of fouling during the operational process.^165^ Most importantly, the working temperature of MD usually ranges from 320 to 350 K, which is much lower than the conventional distillation technologies (e.g., boiling point). Thus, it allows various sustainable low‐grade heat sources such as solar, geotherm, and waste heat from industrial plants as the energy input to driven MD process.^166^ Taken these into consideration, MD has been regarded as a potentially promising candidate to be applied for low‐energy water purification.

**Figure 10:**
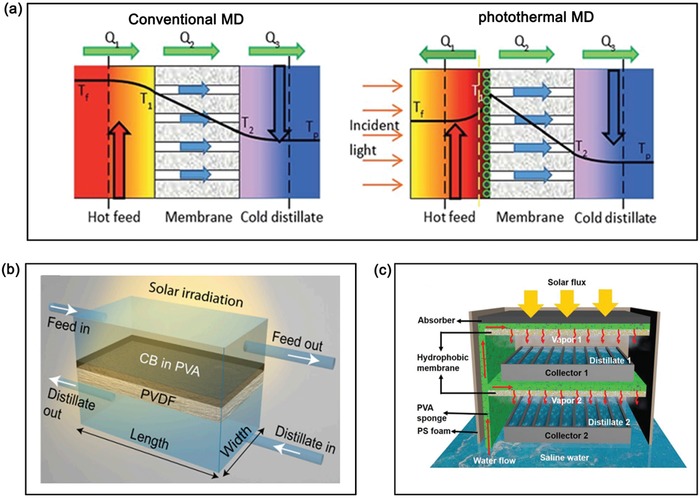
Photothermal‐assisted MD for water purification. a) Schematic illustration of conventional MD and novel photothermal MD with localized heating at the membrane surface. Reproduced with permission.^169^ Copyright 2017, Royal Society of Chemistry. b) Schematic of the photothermal MD module. Reproduced with permission.^170^ Copyright 2017, National Academy of Sciences. c) Schematic illustration of solar‐thermal membrane distillation system with effective cooling strategy and recycling the latent heat. Reproduced with permission.^171^ Copyright 2018, Wiley‐VCH.

Despite great opportunity to solve the water–energy nexus, conventional MD is usually faced with some inevitable limitations, even using low‐grade heat as hot source. On one hand, the utilization efficiency of thermal energy is very low due to numerous heat losses. When there are no recirculation or heat recovery systems used in MD process, most thermal energy would be lost through hot feed discharge. In the previous study, the heat losses of hot feed water reached up to 95% only in the presence of single pass water recovery.^167^ Although the introduction of multiple recirculation and heat recovery measures can reduce heat losses to some extent, these have to add the complexity and capital investment of MD module. On the other hand, the surface temperature of membrane on the side of hot feed water is lower than that of the bulk water, denoted as temperature polarization (**Figure** **10**a), because of the removal of latent heat by water evaporation of membrane surface. As a result, the decreased cross‐membrane temperature difference would lead to a sharp reduction of net driving force,^168^ so that the overall performance of MD drastically falls down. Actually, the membrane surface temperature on the side of hot feed water is highly dependent on the net driving force through membrane, rather than the temperature of entire hot feed water. Inspired by the interfacial heating water evaporation (in Section 3.1), localized heating at the membrane surfaces has provided an appealing solution to address these concerns, especially temperature polarization, so as to obtain highly efficient thermal utilization and high‐performance MD (Figure 10a).

Tremendous efforts have been recently devoted to developing various strategies to fulfill the localized heating during MD process, including joule heat^172^, ^173^ and photothermal effect.^103^, ^169^, ^170^, ^174^ Among them, the incorporation of photothermal materials and MD technology has attracted intensive attention because of its low‐energy and low carbon footprint features. Up to date, there are two typical approaches to implant photothermal materials into MD module: blending method and postfunctionalization. In 2016, Curcio and co‐workers first demonstrated the successful imbedding of thermoplasmonic Ag nanoparticles into microporous PVDF membranes as photothermal module during the nonsolvent‐induced phase inversion process.^174^ They found that Ag nanoparticles were able to notably increase the feed temperature at the membrane surface, which was 23 K greater than the bulk water under the illumination of UV light at 366 nm. As a consequence, the transmembrane flux could increase by 11‐fold at a loading content of 25% Ag nanoparticles, and no occurrence of salt leakage confirmed the distillate with good quality. However, it still remains some drawbacks: a) Ag nanoparticles exhibited low utilization efficiency of solar (i.e., only UV light), and b) photothermal Ag nanoparticles were uniformly distributed throughout PVDF membrane, resulting in a narrowed temperature difference between two membrane sides to decrease the net driving force. In 2017, to alleviate these concerns, a bilayered MD membrane structure was reported, consisting of upper composite porous film of PVA and CB nanoparticles as the photothermal layer and bottom commercial PVDF membrane as the hydrophobic barrier (Figure 10b).^170^ Such configuration design makes the difference of feed and distillate temperatures stabilize in the range of 14–20 °C, insuring a steady net driving force cross the membrane. Moreover, the MD module with an active area of ≈1 × 1 m^2^ can produce ≈4 L day^−1^ under 8 h of sunlight in the summer (700 W m^−2^) without any form of heat recovery, which is enough to meet the basic drinking water requirements of two persons. However, the thickness of photothermal coatings in this bilayered membrane is up to 25 ± 7 µm, and a large number of photothermal CB nanoparticles were still imbedded within the PVA film. To completely address the issue of temperature polarization, the thickness of the photothermal coatings needs to be further reduced.

Surface modification method with conformal function has been widely used to immobilize the photothermal materials on the hydrophobic MD membrane surface. The primary principle of this route is to enhance interfacial adhesion between the photothermal layer and the bottom hydrophobic membrane, as well as remain unchangeable hydrophobicity of MD membrane. For instance, a novel photothermal MD module was constructed by two steps: deposition of Mxene on the hydrophobic PVDF membranes via vacuum‐filtration process, followed by a dip coating of PDMS. The results have found that MXene coating can achieve a reduction of 12% of heater energy input per unit volume distillate, as well as endow MD module with outstanding antifouling functionalities even under 21 h continuous filtration of a working feed (200 ppm BSA and 10 g L^−1^ NaCl).^175^ However, aforementioned MXene coating struggles to low adhesion to the PVDF membrane. In the same year, PDA coating, strong interfacial adhesion with various substrates, was used as the photothermal layer to modify PVDF membrane.^103^ The PDA‐modified membrane displayed the best energy efficiency among current photothermal membranes (45%) and the highest water flux (0.49 kg m^−2^ h^−1^) using DCMD system under 0.75 kW m^−2^ solar irradiation, which is much higher than that of no photothermal layer (0.09 kg m^−2^ h^−1^).

In spite of the success of above‐mentioned photothermal MD, the position of photothermal materials is still located under the feed water, and thus water media can act as a physical barrier to decrease the incident light intensity via reflection or refraction. Besides, resembling the interfacial heating system of water evaporation (in Section 3.1), a part of heat originated from the photothermal effect would warm up the bulk feed (i.e., upward transfer), leading to a low thermal efficiency. By optimizing the configuration of photothermal MD module, Zhou and co‐workers successfully realized the separation of photothermal materials (on the top of MD device) and bulk water and introduced multiple cycles of latent‐heat recovery and effective cooling technologies (Figure 10c).^171^ As a consequence, the two‐level distiller could achieve distillate productivity as high as 1.02 kg m^−2^ h^−1^ with a solar efficiency of 72% upon one sun illumination. Most importantly, the outdoor experiments exhibited a water productivity of 3.67 kg m^−2^ and a salt rejection over 99.75% even in one cloudy day (from 7:45 a.m. to 4:45 p.m. on 26 July 2017). To recap, how to design optimal and integrated configuration is very crucial to improve the performance of next‐generation MD module.

### 3.3 Photothermal‐Assisted Crude Oil Cleanup for Water Purification

Over the past decade, water pollution has been considered as one of the greatest concern along with the fast development of society, especially from those frequent oil spill accidents and industrial oily wastewater discharge. In order to eliminate oil spills for water purification, a great deal of human and financial resources have been costed to develop a variety of materials and treatment technologies. From the perspective of practical applications, two kinds of treatment technologies have received intensive attention as potential candidates: 1) physical adsorption using hydrophobic/oleophilic porous materials,^176^, ^177^, ^178^, ^179^ and 2) oil/water separation based on porous membranes or meshes with superhydrophilic–underwater–superoleophobic property.^180^, ^181^ However, the major problem of above‐mentioned separation protocols is only effective to light oil with low viscosity (<100 mPa s at room temperature),^182^ whereas it does not work for heavy crude oil with a high viscosity from 10^3^ to 10^5^ mPa s at room temperature.^183^ These viscous crude oils have poor mobility and low diffusion rate through the inner pore of porous materials, resulting in low adsorption or separation efficiency. In addition, due to the strong adhesion, the viscous crude oils can tightly cling on the materials surfaces to form serve fouling and even block the inherent inner pores, leading to a significant reduction or even complete destruction of separation performance. Thus, the primary principle of crude oil cleanup is to design and exploit an easy‐to‐implement, low‐energy, and highly efficient route to reduce the viscosity and adhesion of crude oil.

It has been well known that the viscosity of crude oil declines as the increase of its temperature. Thus, in situ heating shows enormous hope to clean up viscous crude oils from water surface for water purification. In 2017, Yu and co‐workers demonstrated that a Joule‐heated graphene wrapped sponge was able to successfully remove crude oil from water surface, because Joule heat generated from electric power can significantly decrease the viscosity of crude oil so as to improve the oil‐diffusion coefficient.^183^ Although it is the first time to adopt in situ heating strategy to realize the cleanup of viscous crude oil, the energy‐consuming feature (i.e., a lot of extra electric power supplies) greatly hampers its practical application, especially in some offshore areas or remote off‐grid areas. Therefore, it is highly desired to develop a new candidate to replace the Joule‐heated method for achieving crude oil spill remediation for low‐energy water purification.

**Figure 11:**
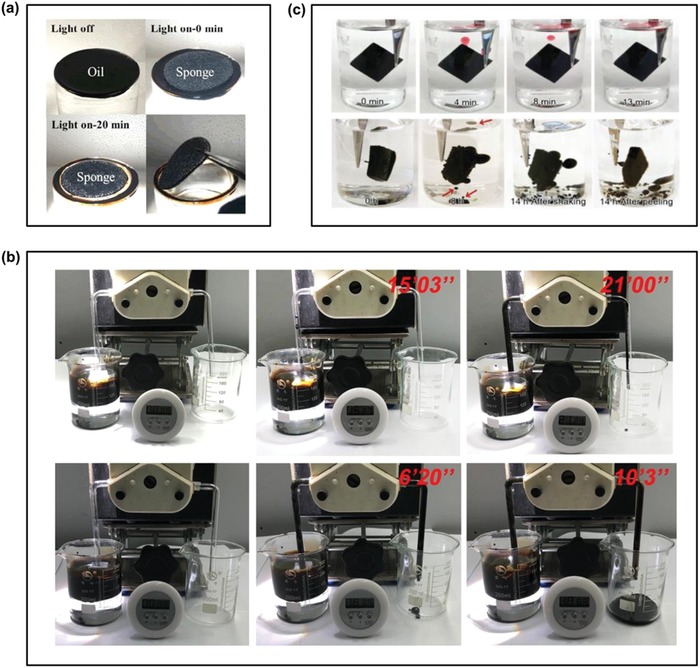
Photothermal‐assisted crude oil cleanup for water purification. a) Digital pictures of the intermittent cleanup process of heavy oil spill using CNTs/PDMS‐modified polyurethane (PU) sponges under one sun illumination. Reproduced with permission.^75^ Copyright 2018, Royal Society of Chemistry. b) Continuous cleanup of high viscosity crude oil spills from the water surface using PDMS‐coated sponge (up) and PDA/PDMS‐coated photothermal sponge (down). Reproduced with permission.^106^ Copyright 2018, Royal Society of Chemistry. c) Digital pictures of the release process of mineral oil (up) and bitumen (down) from PNIPAm/PPy‐modified melamine sponges underwater at 22 °C. Reproduced with permission.^186^ Copyright 2018, Wiley‐VCH.

Resembling above solar‐driven technologies, tremendous efforts have been recently paid to directly utilize photothermal effect to obtain solar‐driven self‐heating function to further reduce the viscosity of crude oil. For example, PPy–polyethylene glycol (PEG)‐polymerized octadecylsiloxane (PODS) coated sponges were fabricated via two‐step surface modification strategy, which takes full advantage of the photothermal property of PPy to heat the viscous oil, as well as the hydrophobic PODS to improve the adsorption capacity of sponges to viscous oil.^184^ The results indicate that the surface of PPy–PEG–PODS coated sponges can reach an equilibrium temperature at 45 °C after 20 min solar irradiation, and such a high temperature would significantly decrease the viscosity of viscous oil. As a result, its adsorption capacity to viscous oil was increased by 32.6%, and the adsorption time was decreased by 40.0% compared with conventional sponges. Similarly, a one‐step method was proposed to modify polyurethane (PU) sponge by incorporating CNTs onto the PU skeletons using commercial polydimethylsiloxane (PDMS) as glue binder.^75^ Thanks to the excellent photothermal effect of CNTs, the oil‐sponge interfacial temperature has a sharp rise to nearly 40 °C after 6 min, corresponding to a reduction of its viscosity from 10^5^ to 4106 mPa s. This photothermal sponge has the ability of adsorbing almost of all the heavy oil (2.5 g) during 20 min light illumination (**Figure** **11**a) and could remain satisfied reusability and recyclability during five cycles of adsorption and desorption experiments. To further improve the adsorption rate of crude oil, Hu's group used modified natural wood with aligned channels as the photothermal sorbent to obtain a high crude oil adsorption rate of 1550 mL m^−2^ in 30 s under one sun irradiation, which is ten times faster than that of aforementioned example.^185^ However, their major bottleneck is that the adsorbed crude oil required to be frequently squeezed out from the inner pores of sponges once the maximum adsorption capacity was reached. Thus, this discontinuous cleanup operation significantly impedes its large‐scale application. Taken this into consideration, we reported a solar‐driven self‐heating sponge comprised of PDA coating as the photothermal layer and PDMS coating as the hydrophobic layer, and was able to be equipped with a peristaltic pump to act as a “self‐heated vacuum cleaner” for highly efficient continuous clean‐up of high viscosity crude oil from water surface (Figure 11b).^106^ The adsorption capacity of self‐heating sponges ((1.29 ± 0.37) × 10^6^ g m^−3^) was nearly 11 times higher than that of without self‐heating feature ((1.13 ± 0.11) × 10^5^ g m^−3^), and more and more crude oils were continuously drew from the water surface with prolonged operation time. Unfortunately, the light‐to‐heat efficiency of these photothermal sponges would be affected under continuous operation process because of inevitably residual crude oil on the sponge surface. Most recently, a novel photothermal sponge could fast adsorb heavy oil under sunlight irradiation and passively release the adsorbed heavy oil underwater at room temperature without any extra operations (Figure 11c).^186^ This functional sponge was rationally designed and fabricated by combining the excellent photothermal effect of PPy with thermoresponsive property of poly(*N*‐isopropylacrylamide) (PNIPAm), endowing modified sponge with oleophilicity/hydrophobicity under sunlight and hydrophilicity at room temperature. Thanks to this reversible transition of hydrophobicity and hydrophilicity, the heavy oil recovery was up to 87%, ensuring a minimum influence of residual heavy oil on the light‐to‐heat efficiency.

Solar‐driven photothermal effect is first proposed and applied in the cleanup of crude oil at 2018, but this emerging filed has attracted increasing research interest. This appealing application is able to not only solve the problem of crude oil spills for water purification, but also immensely broaden and enrich the application scope of photothermal materials. Until now, these stat‐of‐the‐art photothermal adsorption materials are usually prepared by surface modification strategies, and the common configuration design is that the photothermal layers are covered or embedded into the upper hydrophobic layers. As a consequence, the hydrophobic layers have to keep high transparency to maximize the incident light losses, and this requirement limits the selection scope of hydrophobic materials to some extent. In general, the ideal adsorption materials need to possess these characters: large surface area, high thermal conductivity and good manipulation processability, excellent photothermal effect, robust hydrophobic property, and fast passive release ability. Note that photothermal sponges generally have better possess high thermal conductivity to enable the generated heat to fast transfer to the interface of crude oil and sponges via heat conduction, so as to rapid reduce the crude oil viscosity and further improve diffusion coefficient. Therefore, high thermal conductivity plays a significant role in the photothermal‐assisted crude oil cleanup, which is very different from the above‐mentioned thermal management during the interfacial water evaporation system. Unfortunately, polymer sponges used in above examples have a low thermal conductivity, whereas some metal‐based sponges usually exhibit thermal conductivity yet poor flexibility and manipulation processability. Thus, how to balance the thermal conductivity and flexibility of photothermal sponges is very crucial. Although there still remains great challenge, it leaves huge freedom to exploit and design the optimal materials and devices to obtain highly efficient crude oil cleanup for low‐energy water purification.

### 3.4 Photothermal‐Enhanced Photocatalysis for Dye Degradation

In the past two decades, photocatalytic technology has been considered as the green and promising candidate to be applied in environmental remediation field,^14^, ^187^, ^188^ which has the ability of totally decomposing organic pollutants into carbon dioxide and water under the assistance of solar. It has been well known that the photocatalytic efficiency is highly dependent on the generation of reactive oxide species (ROS) originated from the reduction and oxidation reaction of photoexcited electrons and holes, respectively. Thanks to strong oxidability of ROS, the great advantage of photocatalytic technology is able to completely degrade dye contaminants, rather than enriching the dyes from low concentration to high concentration like conventional physical adsorption^189^ and membrane separation technologies.^190^, ^191^ Recently, some previous reports have found that the photothermal materials are capable of subtly integrating into catalysts or photocatalysts to significantly enhance their catalytic efficiency via photothermal effect.^192^, ^193^

**Figure 12:**
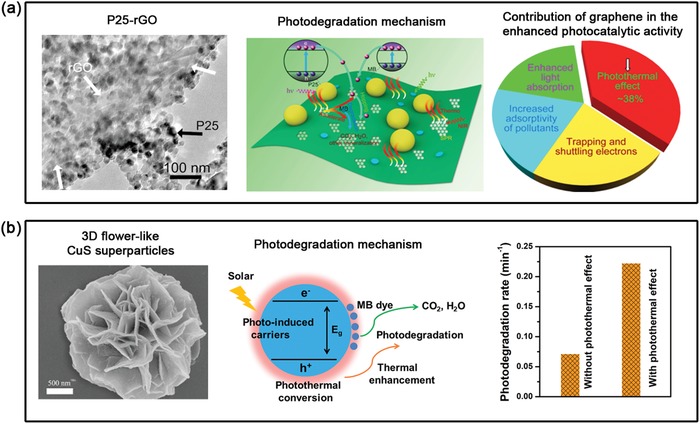
Photothermal‐enhanced photocatalysis for dye degradation. a) SEM image, photodegradation mechanism, and contribution of graphene in the enhanced photocatalytic activity using P25/rGO as photocatalyst. Reproduced with permission.^194^ Copyright 2014, American Chemical Society. b) SEM image, photodegradation mechanism, and photodegradation rate of CuS nanoparticles as photothermal photocatalyst. Reproduced with permission.^195^ Copyright 2018, Wiley‐VCH.

A typical example presented in 2014, Chu and co‐workers first demonstrated that the photothermal effect of rGO plays an important role in photocatalytic performance, and its contribution was as high as ≈38% in the entire degradation action of MB contaminants (**Figure** **12**a).^194^ As the surface temperature of rGO increased after NIR irradiation, the photoexcited electrons could obtain extra energy to fast move on the hot rGO sheets, resulting in a high efficiency of charge transfer. As a result, the recombination rate of photoexcited electron–hole was significantly decreased, which could benefit in the formation of ROS to further lead to higher photocatalytic performance than that of no photothermal effect. In addition, as briefly mentioned in Section 3.1, water evaporation can significantly boost RhB adsorption to the rGO‐based solar absorber surface with the guidance of upward vapor flow,^158^ which would cause device fouling to deteriorate water evaporation performance. When anatase TiO_2_ nanoparticles were deposited on above rGO‐based solar absorber surfaces, the degradation efficiency of RhB increased from 60% (only TiO_2_) to more than 95%. It was because that high temperature generated by the photothermal effect of rGO could not only promote fast enrichment of RhB around the TiO_2_, but also offer enough kinetic energy for the rapid transportation of photoexcited electrons for improving photodegradation rate. Additionally, Sun and co‐workers proposed a gold‐nanorod‐decorated TiO_2_ rambutan‐like microsphere, and this unique 3D morphology configuration could achieve the multireflection of photons to enhance the light absorption efficiency.^12^ As a consequence, it would tremendously facilitate the formation of hot electrons in Au nanorod through the localized surface plasmonic resonance, and their yield was as high as 2.5 times compared with the nanowires. Actually, as‐formed hot electrons were used for producing not only numerous ROS to initiate the degradation reaction of RhB, but also heat energy to promote degradation efficiency. Therefore, this photothermal photocatalysis displayed enhanced photocatalytic performance. Most recently, a 3D flower‐like CuS photocatalyst was prepared and exhibited broadband optical‐absorption properties, which has the ability of directly acting as photothermal photocatalyst without the composite of photothermal materials (Figure 12b). Surprisingly, the photodegradation rate of MB was found to be increased by ≈212.7% assisted by robust photothermal conversion effect.^195^ Apart from the enhanced mobility rate of photoexcited electrons, they proposed that photothermal‐increased temperature could also improve the photoexcitation efficiency (i.e., the generation of photoexcited electrons and electrons), as well as accelerate photodegradation rate via an endothermic process.^195^

As we can see from the above examples, the photothermal effect can effectively improve the photocatalytic degradation rate and inject new vitality into photocatalysis based water purification technologies. Nevertheless, the enhanced mechanism still remains elusive, and many researchers possess different perspectives. Thus, there are no fundamental principles to guide us to design and develop high‐performance photothermal photocatalysts for water purification. Actually, the major bottleneck to clearly elucidate the enhanced mechanism is that it is very difficult to distinguish the contribution of photothermal effect from other factors. For instance, many previous studies have demonstrated that the photocatalytic activity of various photocatalysts is able to be tremendously improved when they are composited with graphene and its derivatives, due to their merits of high specific surface area and unique electronic properties.^196^, ^197^, ^198^, ^199^ Particularly, graphene has the capacity of shifting the Fermi level to decrease the conduction band potential, as well as acting as an electron‐acceptor material to effectively hinder the electron–hole pair recombination. Thus, these inherent natures of graphene can have significant contribution on the enhanced photocatalytic activity, making it difficult to separate from the contribution of photothermal effect.

### 3.5 Photothermal‐Assisted Water Harvesting from Air

Atmospheric water in the form of vapor and droplets preserves almost 13 billion tons of fresh water, equivalent to ≈10% of all fresh water in lakes.^24^ Consequently, harvesting water from air has emerged as another promising approach to address the water shortage issue, especially in the arid and landlocked regions. Although there have been much efforts to collect water from air by fog capture and active refrigeration, high ambient relative humidity (RH) or alternative electric energy is necessary in these processes.^200^, ^201^ In recent years, photothermal‐assisted water harvesting method has been demonstrated to be an idea candidate to produce clean water from dry air (i.e., RH is even lower than 20%) in the off‐grid area. In this approach, photothermal water sorbent first captures water from air and then uses solar‐driven photothermal effect to generate heat to facilitate the release of the absorbed water. Thus, the ideal water sorbents should be able to adsorb large amount of water from air even at low humidity and fast release water at a relatively low temperature (60–80 °C), which can be available by photothermal materials under regular or even weakened sunlight intensity.

**Figure 13:**
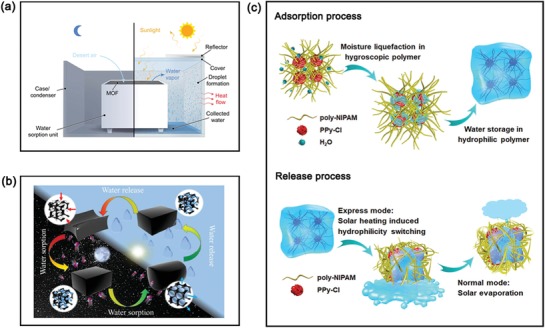
Photothermal‐assisted water sorbents for harvesting water from air. a) Schematic of the water harvester based on MOF‐801‐based water sorbent. Reproduced with permission.^204^ Copyright 2018, American Association for the Advancement of Science. b) Schematic illustration of the water harvesting cycle of CaCl_2_ based water sorbent. Reproduced with permission.^208^ Copyright 2018, American Chemical Society. c) Schematic of water adsorption and release process based on PNIPAM and PPy–Cl hybrid hydrogel. Reproduced with permission.^209^ Copyright 2019, Wiley‐VCH.

Metal–organic frameworks (MOFs), constructed from metal nodes and organic linkers, have emerged as promising materials for water harvesting from air, owing to their high surface area and adjustable pore size and surface chemistry. In 2017, Yaghi and Wang and co‐workers first demonstrated a porous MOF (MOF‐801, [Zr_6_O_4_(OH)_4_(fumarate)_6_]) exhibited superior ability to capture water from the atmosphere at low humidity levels (as low as 20%) by using low‐grade heat from natural sunlight.^202^, ^203^ In this device, the water adsorbent layer was fabricated by infiltrating MOF‐801 powders into porous copper foam, while the solar absorber side was coated with graphite or pyromark painting to ensure the MOF‐801 with high temperature (i.e., 80 °C) to desorb water from. Surprisingly, 2.8 L water per kilogram of MOF daily can be delivered at low humidity levels without any energy input. However, the separation of photothermal materials and MOF‐801 brings a huge obstacle to utilize solar‐driven heat to obtain the total release of absorbed water within the pores of MOF‐801, because of low thermal utilization efficiency. Subsequently, to solve this issue of thermophysical property, an all‐in‐one photothermal water absorber was designed through blending 33% (wt%) of nonporous graphite into MOF‐801 layer, and its water harvesting performance was evaluated under the real desert climate as shown in **Figure** **13**a.^204^ The results found that this device can produce 100 g of water per kilogram of MOF‐801 per day‐and‐night cycle after carefully modifying the condenser and the title angle to the sunlight. Furthermore, more than twice the amount of water can be delivered, when MOF‐801 was replaced by MOF‐303, a porous material with similar water adsorption isotherm but higher water uptake. Up to date, many other MOFs such as CAU‐10, UIO‐66, MOF‐841 and aluminum fumarate have been developed and used for atmospheric water harvesting in consideration of water adsorption isotherm.^24^, ^205^, ^206^, ^207^ Recent report has demonstrated that optimized water uptake ability will be achieved if the pore diameter of MOFs is above the critical diameter of water capillary action.^207^ Thus, the flexible design and synthesis of MOF materials endow it with great possibility to achieve the satisfactory water sorption properties.

Some anhydrous salts with strong hydration ability are also employed as promising atmospheric water sorbents, and they are usually inexpensive and widely commercially available compared with MOF. Recently, Wang and co‐workers demonstrated three anhydrous salts, such as copper chloride (CuCl_2_), copper sulfate (CuSO_4_), and magnesium sulfate (MgSO_4_), were capable of act as efficient water sorbent because of better water absorption/release capability and chemical/physical stability compared with other 11 kinds of common salts.^210^ Subsequently, a bilayer water collection device was designed with the top layer of the photothermal CNT layer and the bottom layer of a salt‐loaded water vapor adsorption layer. Under low humidity (10–35%), this bilayer device was capable of harvesting 20% liquid water of their own weight and releasing water under one sun irradiation (photothermal temperature is 65 °C after 200 s). Actually, in comparison with aforementioned physical stable salts, deliquescent salt like CaCl_2_ has a higher affinity with water and is able to absorb water vapor as many as 5–6 times its own weight. Nevertheless, the captured water vapor eventually dissolves the salt to form a water solution, triggering troubles in handling and engineering design for water harvesting. To overcome this obstacle, CaCl_2_ was assembled into a hydrogel network to maintain the salt in a solid form even after absorbing a large amount of water.^208^ As shown in Figure 13b, the deliquescent CaCl_2_ endows the composite hydrogel with outstanding water sorption capacity even in low humidity air. With the help of CNT as photothermal material, the homemade prototype device with 35 g dry hydrogel can deliver 20 g of freshwater within 2.5 h under outdoor conditions.

It has been well known that the water content of hydrogel is up to 80–90%, and thus dry hydrogel has great potential as the fire‐new water sorbent. Recently, a super moisture‐absorbent gel, containing hygroscopic polypyrrole chloride (PPy–Cl) penetrating in hydrophilicity‐switchable polymeric network of PNIPAM, was demonstrated to possess a high‐efficiency water production in a broad range of RH.^209^ The synergistic effect of PPy–Cl and PNIPAM enables a high saturate water content of 0.7 g per gram of gel at a RH of 30% (Figure 13c). What's more appealing, the photothermal effect of PPy–Cl can trigger the stimuli‐responsive behavior of PNIPAM from hydrophilicity to hydrophobicity, which is very beneficial for speeding up the water release from hydrogel network. Similarly, Tan and co‐workers demonstrated a superhygroscopic hydrogel was capable of absorbing water from sea water (highly RH) by 420% of its own weight.^211^ Furthermore, the concentration of salt ions (Na^+^, K^+^, Ca^2+^, and Mg^2+^) within the collected water was less than 2 ppm, exhibiting superior desalination ability without additional energy input. These outstanding works pave the way toward the photothermal‐assisted water harvesting from air. It is believed that water production in desert climates can move one step closer to practical application by adopting suitable water sorbent materials and solving the device‐engineering problems.

## 4 Conclusions and Outlook

Solar‐driven photothermal strategy has become one of the most popular candidates to achieve low‐energy water purification/harvesting and address the water–energy nexus over the past decade. Within the framework of this review, we present the recent progress of photothermal materials from fundamental mechanism, materials selection and optimization, and devices design principles to current advanced applications. Among three typical solar absorbers, we mainly highlight carbon‐based solar absorbers, including carbon nanotube and graphene, carbonized materials and black‐carbon‐based coatings, owing to their merits of excellent processability and highly efficient light‐to‐heat conversion. Most importantly, the emerging solar‐driven photothermal applications have been discussed in detail. Photothermal‐assisted water evaporation, MD, crude oil cleanup, and photocatalysis for dye degradation have the capacity of producing freshwater from seawater or polluted water, while photothermal water‐sorption materials can efficiently harvest water from air even under low‐humidity environment. In spite of fast and remarkable developments in both photothermal materials and applications, they still remain a few key challenges, which create exciting future research opportunities as well as call for more research attention.

### 4.1 Challenges

First, there are knowledge gaps in the synthesis and processing of photothermal materials. Up to date, many studies mainly focus on the development of diverse photothermal materials. However, it is very difficult to directly assess their photothermal conversion efficiency just by comparing the literature values of solar utilization efficiency and water evaporation rate, because they have different experimental conditions, devices configurations, and energy efficiency calculation methods. As a result, formulating a uniform and scientific evaluation criterion broad enough to counteract various effects over a wide range of time and length scales for high‐performance solar absorbers is a challenging task. Additionally, the techno‐economic assessment and life‐cycle assessment are another two problems worthy of careful consideration, which directly determine the real scalability of the photothermal materials and devices. Possible route involves exploiting common, abundant, and low‐cost organic biomass or biochar as the solar absorbers to replace the use of photothermal plasmonic metals and semiconductors.

Second, there are many open questions regarding the practical applications of solar steam generators. The previous reports found that most solar steam generator devices exhibited a low water evaporation rate in the range of 1.0–1.5 kg m^−2^ h^−1^ under one sun illumination, and just a few cases can reach or exceed 2.0 kg m^−2^ h^−1^. In aim to improve freshwater productivity, many researchers have adopted the unsustainable strategy to increase light power (even as high as 10 kW m^−2^), which makes solar devices impossible to highly efficient work under real one sun illumination (1 kW m^−2^) or even cloudy day (less than 1 kW m^−2^). Until now, there are only a few examples of solar steam generation device with 100% solar‐to‐vapor energy transfer efficiency, in spite of the implantation of some advanced strategies such as energy gain from environment to improve thermal management,^121^, ^127^ and 3D photothermal structure design to boost utilization of solar energy and minimize heat losses.^114^, ^212^ Thus, it is highly desired to clearly elucidate the underlying mechanism to guide us to design and exploit highly efficient solar‐to‐vapor devices (even 100% solar‐to‐vapor) under one sun or lower light intensity illumination.

Third, photothermal‐assisted membrane distillation and crude oil cleanup technologies just begin to attract research interest, and their fundamental mechanism still remains incompletely understanding. The great bottleneck is the low utilization efficiency of as‐generated heat because of a large number of heat losses. Although the thermal management strategies of solar steam generator can be acted as a reference, there are slight differences about underlying mechanism among these photothermal applications. For example, solar steam generation needs to introduce a barrier to reduce heat transfer to bulk water, while a fast heat transfer to the crude oil interface would benefit in the performance of photothermal‐assisted crude oil cleanup. Thus, it is of vast importance to study and explore theoretical basis for further facilitating new advances in these emerging technologies.

Fourthly, recent breakthroughs of low‐energy water harvesting from air have injected new vitality into the production of freshwater, but it is still in the infant stage. Although diverse high‐performance water adsorption materials (MOF, anhydrous salts, and hydrogel) have been exploited as popular alternatives, the major issue is that the release effect of adsorbed water still remains unsatisfied even under the assistance of solar‐driven photothermal effect. Thus, how to achieve total release of adsorbed water under low‐energy route or even passive release will become research objectives for the next stage.

Last but not least, although the current solar‐driven photothermal applications have given a great hope toward the water–energy nexus, most of them are still in laboratory, and there is a long way to go in large‐sacle applications in the real environment owing to the diversity and complexity in the working conditions. Thus, how to design and develop the photothermal materials/devices with excellent adaptability and robust long‐term stability will play a dominant role in its practical applications.

### 4.2 Future Perspectives

This proof‐of‐concept of solar‐driven low‐energy water purification/harvesting technologies has enough room to further broaden their development and applications from photothermal materials and devices design in the near future. Compared to the conventional desalination technologies, these solar‐driven water purification/harvesting technologies can eliminate the need of complex device configuration, extra electricity input, and high pressure operations, offering the potential to develop highly integrated and portable devices which can be applied in small‐sized water plants and remote off‐grid area. Recently, increasing attention has been devoted to exploring various techniques by further converting the normally wasted resources during the solar‐driven water evaporation process into high value‐added products such as electricity generation, so as to harvest freshwater and energy simultaneously. These wide‐ranging and exciting possibilities will add a new dimension in reconciling the water–energy nexus and addressing the water and energy crisis. To recap, if all the above‐mentioned challenges are addressed, it will pave the way to scalable and feasible commercialization from the laboratory to the real applications. Furthermore, we hope this review can help readers quickly catch hold of the recent progress in photothermal materials and practical applications, and stimulate new thinking on the exploration of advanced technologies to fulfill the blue dream of low‐energy water purification/harvesting.

## Conflict of Interest

The authors declare no conflict of interest.
